# Advances in the clinical application of mesenchymal stem cells for neurological disorders

**DOI:** 10.1186/s13287-026-05229-5

**Published:** 2026-08-18

**Authors:** Mohammad Chand Jamali, Alaa Shafie, Ali Jaber Alqahtani, Rithab Ibrahim Al-Samawi, Hanan Mesfer Alyami, Amal Adnan Ashour, Mohammed Fareed Felemban, Nasrin Mansuri, Faris J. Tayeb, Irshad Ahmad, Mustafa Mudhafar, Salah Ahmed Sheweita

**Affiliations:** 1 Department of Health and Laboratory Sciences, College of Medical and Health Sciences, Liwa University, Al Ain, Abu Dhabi, United Arab Emirates; 2 Department of Clinical Laboratory Sciences, College of Applied Medical Sciences, Taif University, P.O. Box 11099, Taif, 21944 Saudi Arabia; 3 College of Medical and Health Sciences, Liwa University, Abu Dhabi, United Arab Emirates; 4 Department of Clinical Laboratory Sciences, College of Pharmacy, University of Al-Ameed, PO Box 198, Karbala, Iraq; 5 Department of Medical and Surgical Nursing, College of Nursing, Princess Norah Bint Abdurrahman University, Riyadh, Saudi Arabia; 6 Department of Oral & Maxillofacial Surgery and Diagnostic Sciences, Faculty of Dentistry, Taif University, P.O. Box 11099, Taif, 21944 Saudi Arabia; 7 Clinical Laboratory Sciences, College of Applied Medical Sciences, King Khalid University, Abha, Saudi Arabia; 8 Department of Medical Laboratory Technology, Faculty of Applied Medical Sciences, University of Tabuk, Tabuk, Saudi Arabia; 9 Department of Medical Rehabilitation Sciences, College of Applied Medical Sciences, King Khalid University, Abha, Saudi Arabia; 10 Department of Medical Physics, Faculty of Medical Applied Sciences, University of Kerbala, 56001 Karbala, Iraq; 11 Department of Clinical Biochemistry, Faculty of Medicine, King Khalid University, Abha, Saudi Arabia; 12 Department of Biotechnology, Institute of Graduate Studies and Research, Alexandria University, Alexandria, Egypt

**Keywords:** Stem cell therapy, Neural regeneration, MSCs, Translational therapy, Regeneration medicine, Clinical trials

## Abstract

Therapeutic approaches employing mesenchymal stem cells (MSCs) have emerged as a promising avenue for investigating treatments for neurological disorders. This strategy aims to capitalize on the biological properties of MSCs to support the repair of damaged neural tissue and modulate pathological processes. This review provides a comprehensive overview of the current clinical evidence regarding MSC applications in major neurological disorders, including Parkinson’s disease (PD), Alzheimer’s disease (AD), amyotrophic lateral sclerosis (ALS), multiple sclerosis (MS), stroke, spinal cord injury (SCI), and other pertinent disorders. Across clinical studies, MSC administration has generally demonstrated a favorable safety profile and procedural feasibility. However, therapeutic efficacy remains variable and inconsistent across trials. Importantly, differences in MSC sources, manufacturing procedures, delivery routes, dosing strategies, and patient selection contribute substantially to the heterogeneity of reported outcomes. Therefore, the current body of evidence supports the safety of MSC-based interventions, but their clinical effectiveness has not yet been consistently demonstrated. Future progress will depend largely on the standardization of cell preparation and treatment protocols, improved biomarker-driven patient stratification, and rigorously designed large-scale randomized trials.

**Graphical Abstract:**

**Figure:**
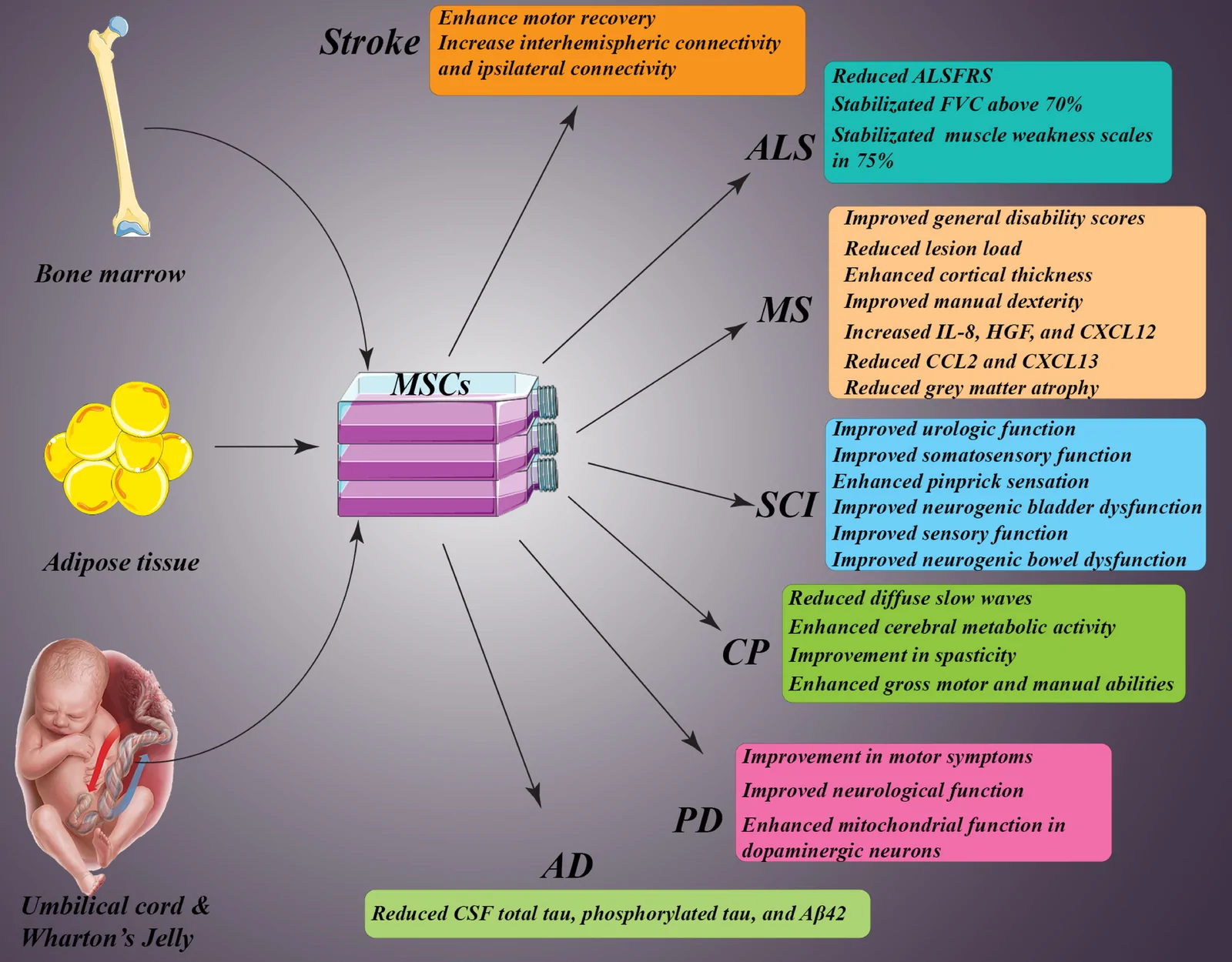

## Introduction

 Neurological disorders, encompassing neurodegenerative diseases, traumatic CNS injuries, and neurodevelopmental abnormalities, constitute a major global health challenge and are among the principal causes of long-term disability and mortality [1, 2]. These conditions are commonly characterized by progressive neuronal dysfunction, persistent neuroinflammatory responses, and the inherently limited regenerative potential of the central nervous system, ultimately leading to permanent neurological impairment and considerable socioeconomic consequences [3]. Although substantial progress has been achieved in the clinical management of these disorders, existing therapeutic strategies remain predominantly palliative, offering symptomatic relief with limited ability to modify disease progression or promote meaningful restoration of damaged neural tissue and function [4, 5].

The pathogenesis of neurological disorders is highly complex and involves the interplay of several interconnected processes, including neuronal damage, dysregulated immune responses, oxidative stress, vascular dysfunction, and insufficient intrinsic repair mechanisms. This multifaceted nature has driven the development of therapeutic strategies that can simultaneously address multiple pathological pathways rather than targeting a single disease component. Among these approaches, cell-based therapies have gained considerable attention owing to their ability to exert broad biological effects, including modulation of the local microenvironment, enhancement of tissue repair, and attenuation of disease progression through diverse and complementary mechanisms [6–8].

Among the various cell-based therapeutic approaches, mesenchymal stem/stromal cells (MSCs) have emerged as particularly promising candidates because of their broad immunomodulatory, anti-inflammatory, neuroprotective, pro-angiogenic, and regenerative capacities [9]. These cells can be readily obtained from several tissue sources, including bone marrow, adipose tissue, umbilical cord, and placental tissues. Rather than replacing damaged cells directly, MSCs primarily exert their therapeutic effects through bidirectional interactions with injured tissues and the release of a wide array of biologically active molecules that influence the local microenvironment [10]. Evidence from numerous preclinical studies, together with findings from early-stage clinical trials, has demonstrated a favorable safety profile and indicates potential therapeutic efficacy in a diverse range of neurological disorders [11].

Despite these encouraging findings, the transition of MSC-based therapies from experimental studies to routine clinical application has been more challenging than originally expected. Although MSC administration has consistently demonstrated a favorable safety profile, therapeutic efficacy has varied substantially between different neurological conditions and even among patients with the same disease. Increasing evidence suggests that this variability is driven by a combination of interrelated factors, including the distinct pathological characteristics of individual disorders, differences in the inflammatory and regenerative status of the tissue microenvironment, heterogeneity among MSC preparations generated from various tissue sources and manufacturing protocols, disparities in administration routes and dosing strategies, and an incomplete understanding of the molecular mechanisms that regulate MSC function, survival, and persistence following transplantation [12].

Concurrently, rapid progress in single-cell technologies, multi-omics analyses, extracellular vesicle research, and biomarker identification has substantially expanded our understanding of MSC biology. These advances have revealed that MSCs do not represent a homogeneous therapeutic platform; instead, they comprise biologically diverse cell populations whose functional properties and therapeutic effects are strongly influenced by the surrounding microenvironment and disease context. Accordingly, the successful clinical implementation of MSC-based therapies will likely depend on the adoption of biomarker-driven patient selection, comprehensive characterization of MSC products, harmonized manufacturing protocols, and the development of disease-tailored therapeutic strategies that bridge mechanistic understanding with clinical application [13, 14].

In this review, we provide a critical appraisal of the current status of MSC-based therapies for neurological disorders by integrating recent mechanistic insights with emerging clinical findings. In addition to summarizing evidence from individual neurological diseases, we explore the common factors that influence therapeutic responsiveness across different conditions. We further discuss the impact of MSC heterogeneity and manufacturing-related variability on treatment outcomes, assess the potential role of emerging biomarkers and precision medicine strategies, and propose a translational framework aimed at facilitating the rational development and clinical implementation of MSC-based therapies for neurological disorders.

## MSCs

In 1968, A. J. Friedenstein was the first to isolate and culture MSCs. By utilizing cells derived from murine BM, Friedenstein found that transferring these cell colonies into semi-syngeneic animals could lead to the development of fibrous tissue and bone. Nevertheless, it wasn’t until several years later that it was recognized that Friedenstein’s findings were attributable to cells with multipotent capabilities [15]. The phrase “mesenchymal stem cells” was introduced by Caplan in 1991, following his research on human BM [16]. As a result of their straightforward isolation, ability to expand, and multipotential characteristics, MSCs have quickly gained popularity as a potential therapeutic option in regenerative medicine. Currently, this area is a prominent research focus being studied for various applications. The International Society for Cellular Therapy (ISCT) has outlined three key criteria to define MSCs. First, MSCs must be able to adhere to plastic culture surfaces and proliferate. Second, MSCs should express cell surface markers CD73, CD90, and CD105, while lacking markers associated with hematopoietic stem cells, such as CD11b, CD14, CD19, CD34, CD45, CD79α, and HLA-DR. Third, MSCs must demonstrate the ability to differentiate into osteoblasts, adipocytes, and chondrocytes under in vitro conditions [17]. MSCs are ideal candidates for cell therapy due to several reasons: (a) They possess stem cell-like properties, (b) They are easily isolated from source tissues, (c) They present fewer ethical concerns compared to embryonic stem cells, (d) They have a lower risk of teratoma formation compared to induced pluripotent stem cells [18, 19], and (e) Their ability to migrate toward damaged tissues through chemoattraction makes them suitable for a wide range of therapeutic applications [20]. Furthermore, MSCs can secrete various bioactive substances such as proteins, growth factors, chemokines, microRNAs (miRNAs), and cytokines, which indicate their potential usefulness in applications [21–25]. Therefore, it is feasible to utilize MSCs for the therapy of tissues from various sources [26–30].

## Mechanisms of action: from cell replacement to dynamic biological modulation

**Fig. 1:**
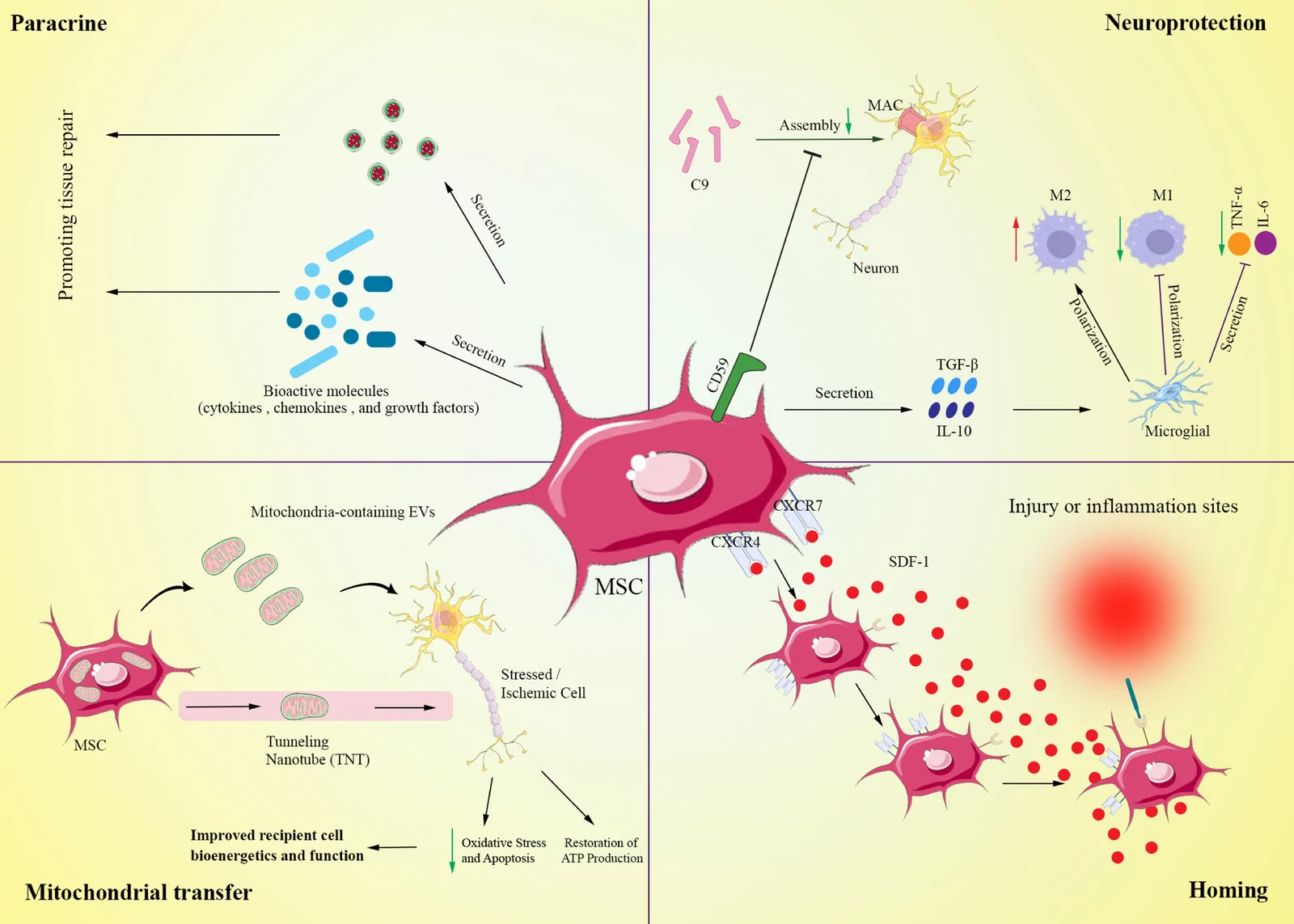
The therapeutic mechanisms of mesenchymal stem cells. MSCs promote tissue regeneration through multiple complementary mechanisms, including paracrine secretion of cytokines, chemokines, growth factors, and extracellular vesicles (EVs); mitochondrial transfer to injured cells via tunneling nanotubes (TNTs) or EVs to restore cellular bioenergetics and reduce oxidative stress; immunomodulation and neuroprotection through inhibition of complement activation and polarization of microglia toward an anti-inflammatory phenotype; and targeted homing to sites of injury or inflammation mediated primarily by the SDF-1/CXCR4/CXCR7 signaling axis

The therapeutic effects of MSCs were initially believed to arise primarily from their ability to engraft within injured tissues, differentiate into neural cell types, and directly replace damaged cells. Nevertheless, subsequent investigations have consistently shown that MSCs exhibit limited long-term survival and only minimal transdifferentiation into neural lineages after administration to the central nervous system. These observations suggest that direct cell replacement is unlikely to be the principal mechanism underlying their therapeutic benefits (Fig. 1) [31, 32]. Instead, current evidence indicates that MSCs act as highly responsive biological mediators that communicate with the injured microenvironment through intricate paracrine signaling networks, immunomodulatory interactions, and metabolic crosstalk. By influencing inflammatory responses, supporting tissue remodeling and angiogenesis, and enhancing endogenous repair pathways, MSCs exert therapeutic effects that are largely determined by the specific pathological and microenvironmental context of each neurological disorder [33, 34].

### Immunomodulation and regulation of neuroinflammation

**Fig. 2:**
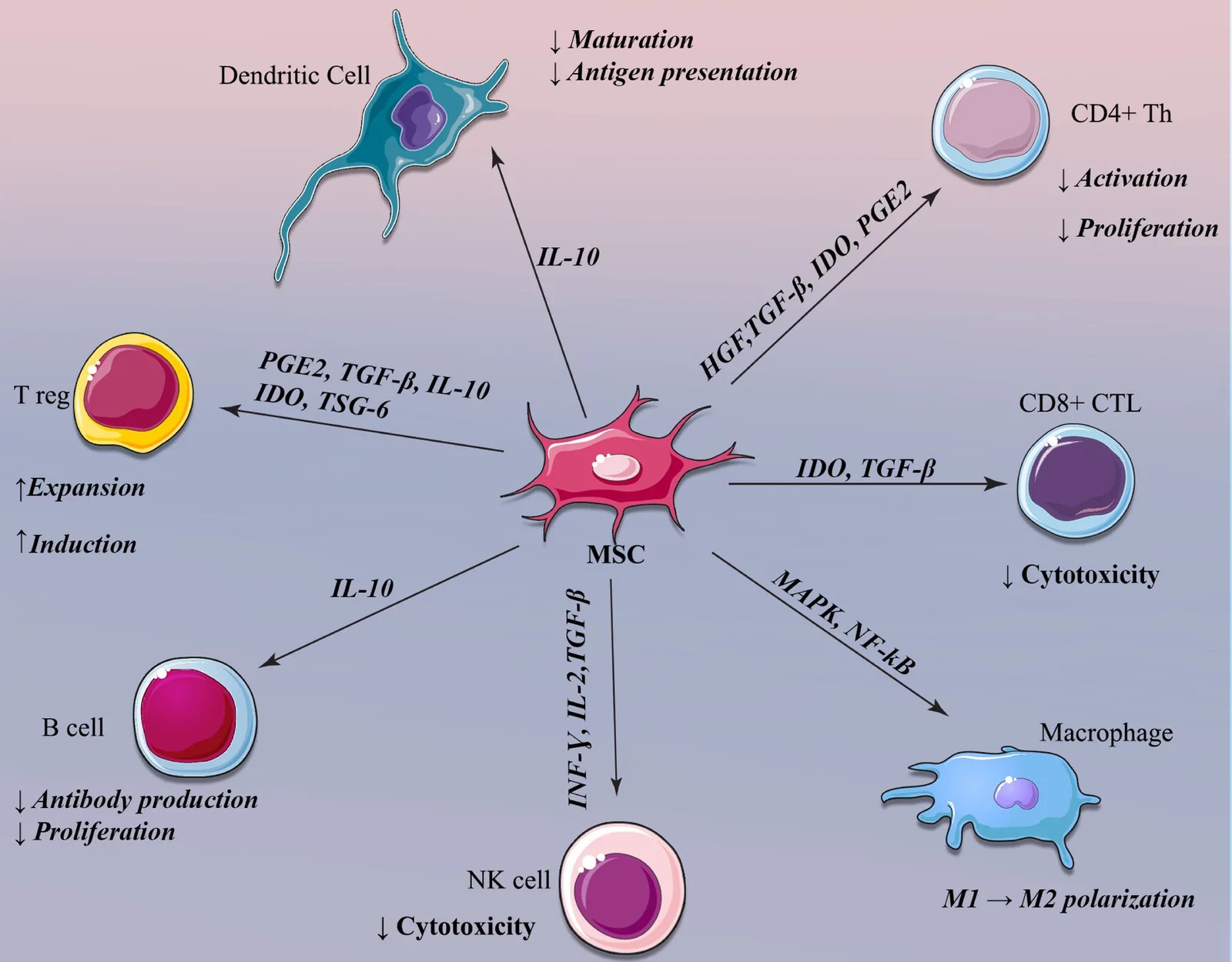
Schematic representation of the immunomodulatory characteristics of MSCs. MSCs regulate immune responses through the secretion of soluble mediators, including TGF-β, IL-10, IDO, PGE2, HGF, and TSG-6. These factors suppress effector immune responses and promote regulatory phenotypes. Specifically, MSCs inhibit CD4 + and CD8 + T-cell activation and proliferation, reduce NK-cell cytotoxicity, suppress dendritic cell maturation and antigen presentation, and decrease B-cell activity. In addition, MSC-derived mediators promote the expansion of Tregs and induce macrophage polarization toward the anti-inflammatory M2 phenotype. Collectively, these interactions contribute to the immunosuppressive and anti-inflammatory properties of MSCs

Neuroinflammation is increasingly recognized as a central driver of neuronal dysfunction and disease progression across numerous neurological disorders. MSCs possess the capability to modulate the immune response in environments characterized by elevated levels of inflammatory cytokines, such as those found in infections, injuries, or immune-mediated diseases. Both preclinical and clinical studies demonstrate that MSCs can effectively downregulate T cell activation and proliferation while promoting the transition of macrophages from the pro-inflammatory M1 to anti-inflammatory M2 phenotypes [35–37]. This adaptive response to inflammatory signals is often described as MSC polarization, whereby MSC behavior is shaped by the surrounding cytokine milieu. These cells can travel to injured tissues following systemic administration, where they provide beneficial effects primarily through mechanisms like immunoregulation and the promotion of angiogenesis [38, 39]. The precise mechanisms underlying the immunosuppressive effects of MSCs remain somewhat unclear. However, it is evident that cellular interactions, facilitated by various factors, play a critical role in this process. In environments with elevated levels of inflammatory cytokines, such as TNF-α and IFN-γ, MSCs secrete a range of cytokines, including TGF-β and hepatocyte growth factor (HGF), and produce soluble factors like indoleamine 2, 3-dioxygenase (IDO), prostaglandin E2 (PGE2), and nitric oxide (NO) (Fig. 2). These mediators contribute to the suppression of T effector cells and promote the expression of FOXP3, CTLA4, and GITR in regulatory T cells (Tregs), thereby enhancing their immunomodulatory capabilities [40–42].

Through their capacity to release bioactive and trophic factors, MSCs have a substantial impact on cellular regeneration and the development of new tissue [43]. MSCs influence immune cells by interfering with their activation, growth, development, and cytolytic function, as well as their production of cytokines or antibodies [44].

CNS and its barriers are abundant in both innate and adaptive immune cells that engage with glial cells during disease processes. Research has demonstrated that the interplay between immune cells and glia is essential for the regenerative capabilities of the CNS [45]. The impact of MSCs on immune cells may play a role in the interactions between these immune cells and glial cells, subsequently affecting the regeneration of the CNS. Therefore, MSC-mediated immunomodulation is considered an important upstream mechanism that can indirectly influence tissue repair processes within the CNS.

Research on microglia provides additional understanding of the function of glial cells and immune cells in the regeneration of the CNS, as microglia can be classified as both glia and immune cells [45]. Research found that MSCs can transform microglia/macrophages from a pro-inflammatory M1 phenotype to an anti-inflammatory M2 phenotype [46]. Scientists found that administering intravenous MSCs in brain led to a decrease in the density of microglia/macrophages and lowered the levels of pro-inflammatory cytokines [47]. A further study examining the therapeutic effects of MSCs through systemic transplantation in a rat model of traumatic brain injury revealed that MSCs decreased microglial activity and enhanced neurogenesis [48]. Immune cells associated with inflammation that have infiltrated the affected region released a range of reactive oxygen species, contributing to programmed cell death in the damaged tissue. MSCs have the potential to mitigate oxidative stress and enhance the expression of the anti-apoptotic Bcl-2 gene within the brain [49].

### Neuroprotection and promotion of endogenous repair

In addition to their immunomodulatory effects, MSCs contribute to neuroprotection through the secretion of a broad spectrum of neurotrophic and pro-angiogenic mediators. Among the most extensively studied factors are brain-derived neurotrophic factor (BDNF), glial cell line-derived neurotrophic factor (GDNF), nerve growth factor (NGF), vascular endothelial growth factor (VEGF), insulin-like growth factor-1 (IGF-1), and HGF [50]. Collectively, these bioactive molecules support neuronal survival, promote axonal growth and synaptic plasticity, enhance vascular remodeling, and create a microenvironment that is conducive to tissue repair and functional recovery following neurological injury.

MSCs exert potent neuroprotective effects by modulating microglial activation and shaping the neuroinflammatory milieu. Through the secretion of anti-inflammatory mediators, particularly TGF-β and IL-10, MSCs suppress the production of proinflammatory cytokines, including TNF-α and IL-6, by activated microglia. Moreover, MSCs promote the phenotypic transition of microglia toward the anti-inflammatory M2 state, thereby attenuating neuroinflammation, limiting secondary neuronal injury, and fostering a microenvironment conducive to neural repair and regeneration [51]. MSCs also contribute to neuroprotection through modulation of the complement cascade. Notably, they express CD59, a complement regulatory protein that inhibits the assembly of the membrane attack complex, thereby limiting complement-mediated neuronal injury [52]. Beyond immunomodulation, MSCs support neural repair through the paracrine release of neurotrophic factors and, to a lesser extent, their capacity for direct differentiation, both of which facilitate axonal regeneration and synaptic remodeling [53, 54]. Furthermore, MSC-derived extracellular vesicles (EVs) and miRNAs serve as important mediators of intercellular communication, regulating neuronal survival, function, and adaptive responses to injury [55]. Emerging evidence also indicates that MSCs can enhance neuronal function by influencing electrophysiological properties and cellular metabolism, further highlighting the multifaceted mechanisms underlying their neuroprotective and regenerative effects.

Several studies have demonstrated that the expression levels of BDNF and NGF are closely associated with the neuroregenerative capacity of MSCs. Increased production of these neurotrophic factors has been correlated with enhanced neuronal survival and greater neurite extension, indicating that they play pivotal roles in mediating the neuroprotective and reparative effects of MSCs within injured neural tissues [56]. Evidence from in vitro studies using rat cortical and hippocampal neurons has further highlighted the importance of BDNF in MSC-mediated neuroprotection. Specifically, depletion of BDNF from the MSC-derived secretome markedly reduced axonal extension, indicating that this neurotrophic factor is a key contributor to the ability of MSC-secreted factors to promote neurite growth and neuronal regeneration [57]. Similarly, the protective effects of BM-MSCs on motor neurons subjected to acetylacetone-induced injury were abolished following the depletion of NGF from the MSC secretome [58]. This observation underscores the critical contribution of NGF to the anti-apoptotic properties of BM-MSCs. Collectively, these findings suggest that both NGF and BDNF represent key mediators of the neuroprotective actions exerted by MSCs. Nevertheless, evidence from other experimental studies has not been entirely consistent, indicating that the relative contribution of these neurotrophic factors may vary depending on the experimental setting and disease context [59, 60].

### Secretome and extracellular vesicle-mediated communication

Growing evidence suggests that the therapeutic benefits of MSCs are mediated predominantly by their secretory activity rather than by direct engraftment and replacement of damaged cells. The MSC secretome represents a biologically complex and dynamic repertoire of secreted components, including soluble proteins, growth factors, cytokines and chemokines, lipids, metabolites, and EVs. MSCs secrete a broad and complex repertoire of bioactive molecules that collectively mediate their immunomodulatory and regenerative functions. Their secretome includes anti-inflammatory cytokines such as IL-10 and TGF-β, as well as IL-6, SDF-1/CXCL12, numerous growth factors, and diverse chemokine ligands. Through the coordinated action of these soluble mediators, MSCs regulate immune responses, support tissue repair, promote angiogenesis, and facilitate the restoration of tissue homeostasis following injury [61].

The MSC secretome consists of a diverse and heterogeneous array of bioactive constituents, encompassing EVs, predominantly exosomes and microvesicles, as well as numerous soluble mediators, including cytokines, chemokines, and growth factors. The coordinated actions of these secreted components enable MSCs to influence cellular communication, regulate local immune responses, and promote tissue repair and regenerative processes [62]. The molecular components of the MSC secretome is highly diverse and functionally complex, comprising a broad spectrum of bioactive molecules, including proteins, lipids, metabolites, and nucleic acids. Through the synergistic interactions of these components, the secretome exerts multifaceted biological effects that influence intercellular communication, immune regulation, and tissue repair mechanisms [63].

Among the various constituents of the MSC secretome, EVs have attracted particular interest because they serve as important vehicles for intercellular communication by transporting biologically active cargo, notably microRNAs (miRNAs) [64]. Following their transfer to recipient cells, these miRNAs modulate post-transcriptional gene expression and influence a wide range of cellular responses. Studies indicates that EV-associated miRNAs participate in several fundamental biological processes, including the regulation of apoptosis, promotion of angiogenesis, and orchestration of tissue repair and regenerative mechanisms [65].

In experimental autoimmune encephalomyelitis (EAE), intravenous administration of EVs was associated with reduced microglial activation and attenuation of both neuroinflammation and demyelination, ultimately translating into improved functional recovery [66]. Comparable findings were reported in another EAE study in which MSC-derived exosomes were delivered intranasally. Notably, intranasal exosome therapy suppressed CNS inflammation more effectively than MSC administration itself and produced superior improvements in disease severity. Collectively, these observations suggest that MSC-derived exosomes may recapitulate, and in some settings even surpass, the therapeutic effects of their parental cells by efficiently modulating inflammatory responses within the CNS [67].

A growing body of evidence has identified numerous exosomal miRNAs as key mediators of the therapeutic effects exerted by MSCs in neurological disorders. In traumatic brain injury (TBI), MSC-derived exosomes transport miR-26a-5p, which attenuates neutrophil extracellular trap (NET) formation and regulates microglial polarization through the TAB2/JNK/AP1 signaling axis [68]. In models of intracerebral hemorrhage (ICH), exosomes derived from AD-MSCs deliver miR-342-3p, which alleviates neuroinflammation by targeting formyl peptide receptor 1 [69]. Similarly, in ischemic stroke, exosomal miR-146a-5p derived from UC-MSCs suppresses microglia-mediated inflammation through inhibition of the IRAK1/TRAF6 signaling pathway [70].

Comparable mechanisms have also been reported in spinal cord injury (SCI), where exosomal miR-21a-5p promotes autophagy and suppresses pyroptosis by targeting pellino E3 ubiquitin protein ligase 1, while miR-24-3p mitigates tissue damage through inhibition of the MAPK9/JNK signaling cascade [71, 72]. In a sporadic Alzheimer’s disease model, small EVs carrying miR-223-3p directly suppress NLRP3 activation and attenuate neuroinflammatory responses [73]. Furthermore, in models of neuropathic pain, exosomal miR-26a-5p targets Wnt5a, whereas miR-99b-3p enhances microglial autophagy, thereby contributing to pain alleviation [74]. Collectively, these findings underscore the pivotal role of exosomal miRNAs in MSC-mediated paracrine signaling, highlighting their function as highly specific molecular regulators capable of reprogramming inflammatory and regenerative pathways within recipient cells.

### Mitochondrial transfer and metabolic reprogramming

More recently, mitochondrial transfer has emerged as another important mechanism underlying the therapeutic effects of MSCs. Increasing evidence indicates that MSCs can donate healthy and functional mitochondria to damaged cells, thereby restoring cellular bioenergetics and improving cell survival under pathological conditions [75]. This intercellular transfer can occur through several routes, including tunneling nanotubes, EVs, and gap junction-dependent communication. Through these mechanisms, MSCs can directly influence the metabolic state of injured cells and contribute to tissue repair and functional recovery [76].

Despite their heterogeneous etiologies and clinical manifestations, many neurodegenerative diseases share common pathogenic mechanisms centered on mitochondrial impairment and bioenergetic dysfunction. Mitochondrial damage promotes excessive generation of reactive oxygen species (ROS), disrupts intracellular Ca²⁺ homeostasis, and amplifies neuroinflammatory responses, collectively contributing to progressive neuronal injury and degeneration within the central nervous system. Consequently, mitochondrial dysfunction, oxidative stress, and aberrant calcium signaling are increasingly recognized as fundamental drivers in the pathogenesis of neurodegenerative disorders [77–80]. By exploiting the intrinsic regenerative properties of MSCs, mitochondrial transfer or transplantation offers a means of restoring cellular function by replacing damaged or dysfunctional mitochondria with healthy organelles derived from MSCs. The acquisition of functional mitochondria can re-establish cellular bioenergetics, reduce oxidative stress, and improve the viability of injured cells, thereby facilitating the recovery of both cellular and mitochondrial function. These observations have positioned MSC-mediated mitochondrial transfer as a promising therapeutic strategy for mitigating tissue damage and promoting regeneration in neurological disorders [81].

MSCs have been shown to protect neural stem cells (NSCs) from cisplatin-induced toxicity through the transfer of functional mitochondria via actin-based intercellular structures. Notably, this protective effect is further enhanced by the overexpression of mitochondrial Rho GTPase 1 (Miro1), a key regulator of mitochondrial trafficking. The acquisition of healthy mitochondria preserves mitochondrial integrity and bioenergetic function in NSCs, thereby improving cell survival and sustaining neural progenitor populations in vivo. These findings highlight mitochondrial donation as an important mechanism contributing to the neuroprotective and regenerative effects of MSCs [82]. Likewise, the transfer of mitochondria from MSCs to stressed neurons has been shown to enhance cellular metabolism and improve neuronal survival. Similar findings have been reported for iPSC-MSCs, which attenuate hypoxia-induced injury in PC12 cells through tunneling nanotube (TNT)-mediated mitochondrial transfer. The delivery of functional mitochondria restores mitochondrial activity, preserves cellular bioenergetics, and increases cell viability under conditions of metabolic stress. These observations further support mitochondrial donation as a key mechanism underlying the neuroprotective actions of MSCs [83].

Experimental studies in mouse models of AD have demonstrated that mitochondrial transfer from MSCs can significantly improve mitochondrial function within the brain [84]. In parallel, MSC treatment has been associated with reductions in Tau hyperphosphorylation and amyloid plaque burden. Additional findings indicate that MSCs inhibit the deposition of amyloid-β (Aβ) plaques and attenuate microglial activation [85, 86]. In experimental models of Parkinson’s disease (PD), mitochondria derived from MSCs have been shown to exert pronounced neuroprotective effects by attenuating the loss of dopaminergic neurons, reducing microglial activation, and improving motor performance [87].

The therapeutic potential of mitochondrial transfer extends beyond neurodegenerative disorders to include acute neurogenic injuries. In SCI, disruption of mitochondrial quality control contributes to neuronal damage by promoting ferroptosis and exacerbating secondary injury processes. Emerging evidence indicates that MSC-mediated mitochondrial transfer can replenish functional mitochondrial populations and suppress ferroptotic cell death by enhancing mitochondrial fusion and preserving mitochondrial homeostasis [88]. Through these mechanisms, mitochondrial donation may represent an effective strategy for limiting tissue damage and promoting recovery following SCI.

Mitochondrial donation has also emerged as an important mechanism contributing to recovery following ischemic stroke. Studies have demonstrated that UC-MSCs can transfer functional mitochondria to both neurons and endothelial cells, thereby restoring mitochondrial integrity and improving cellular resilience after ischemia-reperfusion injury. This mitochondrial exchange reduces the accumulation of ROS, enhances cell survival, and supports tissue recovery. Interestingly, the efficiency of this process appears to be influenced by the oxidative status of the recipient tissue, suggesting that host-derived ROS signaling may regulate the extent and therapeutic impact of MSC-mediated mitochondrial transfer [89]. Similarly, MSCs have been shown to restore mitochondrial function in cerebral microvascular cells, thereby enhancing cellular bioenergetics and viability following ischemic injury. The recovery of mitochondrial activity promotes angiogenic responses, facilitates vascular repair, reduces infarct volume, and ultimately contributes to improved neurological and functional outcomes [90].

### Homing

The homing of MSCs to sites of tissue injury is a highly coordinated, multistep process that depends on the sequential interaction of adhesion molecules, chemokines, and proteolytic enzymes [91]. Following entry into the circulation, CD44 expressed on the MSC surface interacts transiently with endothelial selectins, including E-selectin, P-selectin, and L-selectin, initiating a rolling process along the vascular endothelium. During this phase, MSCs are exposed to chemotactic signals released by injured tissues, particularly SDF-1/CXCL12. The binding of these chemokines to G protein-coupled receptors expressed on MSCs, most notably CXCR4 and CXCR7, activates intracellular signaling pathways that induce conformational changes in integrins and increase their adhesive capacity [92].

Activated integrins subsequently engage their respective ligands on endothelial cells, resulting in firm adhesion and stable attachment of MSCs to the vascular wall. Thereafter, MSCs secrete proteolytic enzymes, including matrix metalloproteinases (MMPs), which degrade extracellular matrix components and loosen endothelial junctions, facilitating transendothelial migration into the surrounding tissue. Once within the interstitial compartment, MSCs migrate along chemokine gradients toward the site of injury or inflammation, where they exert therapeutic effects through immunomodulatory, neuroprotective, and regenerative mechanisms. This tightly regulated homing process is fundamental to the ability of MSCs to localize to damaged tissues and mediate repair [93].

## The potential function of MSCs in CNS regeneration

MSCs have a remarkable capacity to differentiate into various mesodermal lineages that extend beyond their original tissues [94]. This distinctive trait has positioned MSCs at the forefront of clinical research. Investigations have shown that MSCs can express neuronal and astrocytic markers in both in vitro and in vivo settings, underscoring their potential applications in neuroscience [95, 96].

Furthermore, MSCs are known to release a range of growth factors, including BDNF, NGF, VEGF, GDNF, and IGF-1. These factors collectively facilitate neurogenesis, aid in remyelination, and foster a supportive microenvironment for neuronal survival and repair [97–100]. Through the secretion of these trophic factors and immunoregulatory mediators, MSCs contribute to the modulation of the neural microenvironment, which may support endogenous repair processes.

Consequently, MSCs have been extensively investigated in the realm of regenerative medicine, especially concerning the repair of CNS. Research has predominantly centered on employing MSC-based therapies to tackle CNS injuries resulting from severe trauma, extended ischemia, or neurological disorders. This section summarizes recent clinical progress in MSC-based tissue engineering for CNS regeneration. As indicated in Table 1, numerous studies have investigated the possible role of MSCs in the treatment of various neurological disorders, which will be explored in the subsequent sections.

### Multiple sclerosis

MS is a long-lasting autoimmune inflammatory condition that impacts the CNS. The precise mechanisms underlying MS are not yet fully understood. It is widely believed that autoreactive T cells, which are triggered by either self-antigens or similar external antigens, contribute to the process of demyelination and ongoing nerve degeneration in the CNS. Although there are treatments available that can slow the progression of MS and alleviate some disability in affected individuals, these medications often come with significant side effects and do not restore the neurological functions compromised by the disease [101, 102].

Jamali et al. [103] have conducted research examining the therapeutic efficacy of UM-MSCs in patients diagnosed with MS. The study compared two intrathecal UC-MSC protocols in MS patients: Group A received 2 doses of 150 × 10⁶ UC-MSCs, while Group B received 1 dose of 150 × 10⁶ cells. Both protocols were safe, with no severe adverse events, and improved general disability scores by 6 months. However, Group A demonstrated superior outcomes, including increased CD3 + CD4+ lymphocytes, reduced lesion load, enhanced cortical thickness, and improved manual dexterity and processing speed at 12 months. Group A uniquely suppressed TNF-α, TAP-1, and miR-142, suggesting that two doses may enhance immunomodulatory effects compared to a single dose. These results highlight a potential advantage of administering two doses of 150 × 10⁶ UC-MSCs over a single dose, warranting larger, multisite studies to validate these findings and clarify the role of repeated dosing in MSC therapy for MS. In a longitudinal phase I clinical trial led by Harris et al. [104], 20 individuals diagnosed with progressive MS were recruited. Between 2014 and 2016, participants received three intrathecal injections of autologous MSC-derived neural progenitors (MSC-NPs), administered at a mean dose of 9.4 × 10^6^ cells (target dose: 1.0 × 10^7^ cells). Clinical outcomes demonstrated improvements in the Expanded Disability Status Scale (EDSS) and timed 25-foot walk (T25FW) performance. Additionally, CSF analysis post-intervention revealed a reduction in CCL2 alongside elevated levels of IL-8, HGF, and CXCL12. No treatment-related serious adverse events were reported. However, the study’s generalizability and interpretability were constrained by its limited cohort size, absence of a placebo control group, and lack of blinding, which may introduce potential biases in outcome assessment. In another randomized trial by Harris et al. [105], 54 individuals with progressive MS were divided into two groups: one receiving six intrathecal injections of autologous MSC-NPs (*n* = 27) and the other receiving intrathecal saline placebo (*n* = 27). The treatment group was administered up to 1 × 10⁷ MSC-NPs per dose, delivered every two months over six sessions, while the placebo group followed the same injection schedule with saline. Although no significant overall differences in disability scores (EDSS Plus) were observed, post-hoc analyses hinted at benefits in patients with higher baseline disability (EDSS 6.0–6.5). These individuals exhibited improvements in walking tests, bladder function, and reduced grey matter atrophy on MRI. Biomarker shifts, including elevated MMP9 and reduced CCL2 in cerebrospinal fluid, further supported potential therapeutic effects. The study proposes that repeated IT-MSC-NP administration may stabilize disease progression in advanced MS, urging larger trials to validate these observations and refine treatment protocols.

Studies showed that antigen presentation by B cells plays a critical role in the activation, proliferation, function, and pathogenicity of autoreactive T cells in MS [106]. There is growing interest in therapeutics aimed at B cells [107, 108], namely MSCs, which suppress B cell activation and enhance Bregs [109]. In support of this idea, a recent study by Shokati et al. has shown that transplantation of placenta-derived MSCs decreased CD20/CD19 B-cell markers and increased anti-inflammatory cytokine IL-10, alongside reductions in pro-inflammatory TNFα, IL-6, and IL-17 in patients with secondary-progressive MS [110].

To compare the therapeutic efficacy of intrathecal versus intravenous administration of autologous BM-MSCs, a clinical study was conducted in 48 patients with MS. Results indicated statistically significant improvements in EDSS scores and MRI-derived outcomes across all primary endpoints, with no severe treatment-related adverse events reported. Notably, intrathecal delivery demonstrated superior performance compared with intravenous delivery across specific clinical and neuroimaging metrics, suggesting enhanced therapeutic effects with direct intrathecal delivery. While these findings are promising, the study authors highlighted critical limitations, including the requirement for larger phase III trials to corroborate the results and refine optimal administration protocols for BM-MSC therapy in progressive MS [111].

Various immunomodulatory therapies for MS have demonstrated moderate reductions in serum (neurofilament light chain) NF-L levels [112]. This is likely associated with the inhibition of inflammation and subsequent relapses, thereby reducing neuronal damage. CSF samples from 48 patients in the previous trial by Petrou et al. [113] were analyzed to assess NF-L levels following MSC treatment in individuals with progressive MS. Among the 15 patients evaluated in the MSC-intrathecal group, nine demonstrated a reduction in NF-L levels of more than 50%, compared to five in the MSC-intravenous group and only one in the placebo group, indicating a more pronounced biomarker response with intrathecal administration. Administering MSCs via intrathecal injection places the stem cells closer to the inflamed regions of the CNS compared to intravenous delivery. This may clarify the noted superiority of intrathecal over intravenous treatment in reducing CSF NF-L levels. These findings suggest that MSC transplantation may have neuroprotective effects in patients with MS. The biomarker reductions could have been attributed to “regression to the mean” or the stabilization of MS after significant prior disease activity, which was a potential limitation of the findings. Recently, Petrou et al. [114] conducted another trial to investigate the effect of repeated MSC-NG01 injections on serum biomarkers of neuroinflammation and neurodegeneration in 23 patients with progressive MS. Individuals received 2 to 3 intrathecal injections of MSC-NG01 and were monitored for more than 12 months. Based on their results, serum levels of NFL and GFAP showed a gradual, consistent reduction after MSC transplantation, indicating a strong, cumulative effect of repeated MSC injections on both neurodegeneration and neuroinflammation. In a similar study, the safety and effectiveness of three intrathecal injections of autologous MSCs secreting neurotrophic factors (MSCs-NTF) were examined in 18 patients with non-relapsing progressive MS. Analysis of CSF indicated an increase in neuroprotective factors, including VEGF-A, HGF, NCAM1, follistatin, LIF, and fetuin-A, and a reduction in inflammatory biomarkers, aligning with the proposed therapeutic mechanism. Nevertheless, the study’s open-label design and the absence of a placebo-controlled group pose a risk of expectation bias, potentially affecting the interpretation of efficacy outcomes. Furthermore, biomarker analysis was limited, as the third CSF sample was collected before the final treatment, reflecting only the effects of the initial two treatments [115].

Berard et al. [116] conducted a study on MSC therapy in people with MS. They found that the treatment was safe and possible, but it didn’t lead to long-term cognitive improvement. These findings suggest that the therapeutic benefits of MSCs might be short-lived, potentially indicating limited long-term viability or functional endurance of the transplanted cells in the CNS. These results support the idea that MSCs primarily act through short-lived, paracrine-driven mechanisms rather than through stable engraftment. Furthermore, a phase 2 randomized study investigated the safety, tolerability, and potential efficacy of autologous BM-MSCs in patients with active MS. The trial included 144 patients with relapsing-remitting or progressive MS, who were randomly assigned to receive either an intravenous infusion of MSCs or a placebo, followed by the opposite treatment after 24 weeks. The study’s primary objectives were to assess safety by monitoring adverse events and evaluate efficacy by measuring the number of gadolinium-enhancing lesions (GELs) at several time points. While MSC treatment was well tolerated, with adverse event rates comparable between the two groups, it did not significantly reduce GELs at week 24. Infections were the most common adverse events, and there were nine serious adverse events in the placebo group and none in the MSC group. The findings indicated that, although MSC therapy was safe, it did not impact acute inflammation, as reflected by GELs, suggesting that MSCs may not be effective for treating active MS. Further research is needed to explore MSCs’ potential effects on tissue repair [117].

Collectively across studies, intrathecal delivery generally yields more pronounced biomarker modulation and clinical stabilization than intravenous administration, likely due to improved proximity to inflamed CNS compartments and avoidance of pulmonary sequestration. Repeated dosing strategies, whether using UC-MSCs, BM-MSCs, MSC-derived neural progenitors, or neurotrophic factor–secreting MSCs, tend to produce more sustained immunomodulatory and neuroprotective biomarker shifts, including reductions in NF-L, CCL2, and pro-inflammatory cytokines, alongside increases in trophic mediators. However, objective disability endpoints such as EDSS frequently show modest or subgroup-restricted benefits, particularly in progressive or advanced disease. In contrast, large randomized trials using intravenous BM-MSCs have confirmed safety but failed to significantly reduce acute inflammatory activity, as measured by gadolinium-enhancing lesions.

### Amyotrophic lateral sclerosis

ALS is a rare and fatal neurological disorder that is idiopathic in nature, primarily impacting the neurons that govern voluntary muscle movements in the human motor system [118, 119]. Owing to the progressive nature of ALS, the severity of symptoms escalates over time [120]. The onset of the disease is often subtle, with symptoms progressively developing into noticeable weakness or muscle atrophy [121]. Initial manifestations may include muscle twitching in areas such as the arms, legs, shoulders, or tongue, alongside muscle stiffness (spasticity), muscle spasms, and difficulties with swallowing [122, 123]. The symptoms start off localized, but as the illness advances, they generally disseminate across the body, which often results in difficulty swallowing (dysphagia), trouble speaking (dysarthria), and problems with breathing (dyspnea) [121, 124]. In ALS, respiratory muscle impairment represents the primary cause of mortality in patients with ALS, typically occurring within 3 to 5 years following the onset of symptoms. Unfortunately, despite comprehensive research efforts focused on ALS, there are currently no clinical or preventive interventions available. The sole approved pharmacological treatment, Riluzole, demonstrates a capacity to modify the disease within this context [125, 126], resulting in an extension of patient survival by approximately 3 to 6 months. However, the effectiveness of this treatment can vary significantly among individuals [127].

Given the aforementioned details, exploring novel therapeutic approaches could extend the lifespan of ALS patients. In this context, MSCs have been utilized for several years in both preclinical and clinical investigations aimed at treating individuals with ALS [128, 129]. Syková et al. [130] evaluated the safety and therapeutic potential of intrathecal autologous MSC transplantation in 26 patients with ALS, administering a dose of 15 ± 4.5 × 10⁶ cells. Post-treatment assessments revealed a slowed decline in ALS Functional Rating Scale (ALSFRS) scores, stabilization or maintenance of forced vital capacity (FVC) above 70%, and stable muscle weakness scales in 75% of patients. The procedure was deemed safe, with no significant adverse events reported during or after transplantation. Barczewska and colleagues reported that administering three intrathecal injections of 30 × 10^6^ WJ-MSCs led to improvements in ALSFRS scores [129]. They demonstrated that WJ-MSCs are both safe and effective for individuals with ALS. Conversely, another research group found that intrathecal injection of autologous adipose MSCs did not enhance the clinical symptoms in ALS patients [131]. Their findings revealed elevated levels of protein and nucleated cells in the CSF, and the ALSFRS-R indicated a progression of the disease in all treated individuals. Because of the small patient sample, it might be challenging to evaluate the data and reach a precise conclusion about the effectiveness of intravenous compared to intrathecal transplantation.

Recently, Karimi et al. [132] conducted a study examining the effects of intravenous versus intrathecal transplantation of WJ-MSCs in 12 ALS patients. Although there was no notable improvement in the ALSFRS-R score or overall clinical effectiveness, patients reported positive changes in specific areas, including salivation, swallowing, and speech. Moreover, there was a reduction in muscle tremors and fasciculations, along with an increase in muscle strength. Given the small patient population, it may be challenging to analyse the data and reach a precise conclusion about the effectiveness of intravenous versus intrathecal transplantation.

Siwek et al. explored the safety and therapeutic potential of intrathecal autologous MSCs in sporadic ALS, administering three doses of 10 × 10⁶ cells each over three months. Although the treatment proved safe, efficacy varied: patients with slow-progressing ALS showed no significant change in disease trajectory, whereas those with rapid progression exhibited modest deceleration in decline, though statistical significance was unattainable due to the limited subgroup size (*n* = 8). The authors highlighted challenges in preparing autologous MSCs for fast-progressing cases and proposed allogeneic cell sources as a pragmatic alternative. Despite encouraging signals, the study’s lack of a control group, small cohort, and short follow-up period necessitate further large-scale trials to validate outcomes and assess long-term benefits [133].

Levels of inflammatory markers such as IL-1β, IL-6, IL-8, and TNF-α are significantly higher in patients with ALS, and there is an increase in the number of activated astrocytes and microglia in the affected regions of the disease [134]. Astrocytes and microglia serve crucial roles in neuroinflammatory processes. Microglia are particularly involved in releasing dysfunctional mitochondria, which in turn activate astrocytes (A1 to A2) to propagate inflammation. This cascade contributes to neuronal degeneration and cell death [135]. Alkhazaali-Ali et al. demonstrated a substantial decrease in TNF-α levels in the CSF of ALS patients following repeated BM-MSC transplantation, with a three-month follow-up [136]. Preclinical studies demonstrated that MSC therapy suppresses microglial secretome, leading to a significant decrease in A1-associated gene expression and an increase in A2-associated gene expression in astrocytes [137]. Recent studies have demonstrated that MSC-derived neurotrophic factor (MSC-NTF) cells enhance the expression of neuroprotective markers, including VEGF, HGF, LIF, as well as microRNAs miR-132p and miR-376, in patients with ALS. Additionally, these cells have been shown to reduce the expression of neuroinflammatory markers such as MCP-1 and SDF-1 and to upregulate the microRNA miR-146. These results support the proposed paracrine mechanism of action of MSC-NTFs and the combined effects on both neuroprotection and neuroinflammation [138].

Reis et al. explored the molecular effects of intrathecal autologous BM-MSCs (1 × 10⁶ cells/kg) in ALS by analyzing CSF proteomics before and 30 days post-treatment. The study identified 220 altered proteins post-therapy, with bioinformatics linking these changes to extracellular matrix (ECM) remodeling and cell adhesion pathways, including targets such as APOA1, APOE, APP, C4A, C5, and fibrinogen/plasminogen components (FGA, FGB, FGG, PLG). These results suggest that BM-MSCs may influence neuroinflammatory and coagulation mechanisms in ALS. However, limitations such as a small cohort (8 of 15 participants analyzed), high withdrawal rates, the lack of controls, and short follow-up hinder definitive conclusions. The authors advocate for larger trials to validate these pathways and address practical barriers in autologous MSC therapy, proposing allogeneic alternatives for scalability [139].

Across ALS studies, MSC therapy has consistently proven safe, but its clinical benefits remain unpredictable and often modest. Rather than halting or reversing disease progression, most trials have observed temporary stabilization in select patient subgroups, particularly those with rapidly progressive disease. Intrathecal delivery appears to engage the CNS more effectively than intravenous infusion. What does seem clear is that a single dose may not be enough. Repeated intrathecal injections have been associated with stronger anti-inflammatory effects, including reduced TNF-α signaling and modulation of glial activity, reinforcing the view that MSCs act primarily as immunomodulators rather than regenerative builders. They do not replace lost motor neurons; they briefly shift the immune landscape. Interpreting these findings is complicated by the heterogeneity across every ALS trial. Some studies use bone marrow-derived MSCs, others use adipose or Wharton’s jelly-derived cells. Patients differ in how quickly their disease advances and how disabled they are at enrollment. Until these variables are controlled for or at least consistently reported, drawing broad conclusions will remain a challenge.

### Spinal cord injury

SCI is characterized by traumatic or non-traumatic damage to the spinal cord, resulting in transient or persistent alterations to motor, sensory, or autonomic functions. This condition is associated with a high global incidence, substantial socioeconomic costs, elevated disability rates, and a propensity for earlier onset compared to many chronic disorders [140]. Etiologies of SCI are broadly categorized into traumatic causes, such as high-impact trauma (e.g., motor vehicle collisions, falls, or violence), and non-traumatic mechanisms, including infections, neoplastic processes, degenerative spinal pathologies, ischemia-reperfusion injury, and vascular anomalies [141, 142]. Severe SCI imposes substantial physical, psychological, and socioeconomic burdens on affected individuals and their families, underscoring the urgent need for innovative therapeutic and rehabilitative interventions. Current therapeutic strategies for SCI remain largely confined to intensive rehabilitation, reflecting a critical need for innovative strategies to promote neuroregeneration and functional recovery. The limited efficacy of existing rehabilitative approaches underscores the urgency to develop novel interventions targeting neuroprotection, axonal regeneration, and synaptic plasticity to enhance neurological outcomes in SCI patients.

Research in SCI regenerative medicine explores diverse donor cells, including Schwann cells [143], olfactory ensheathing cells [144], neural stem cells [145], and MSCs [146], to develop therapies. MSCs stand out for their therapeutic potential, immunomodulatory properties, and ease of harvesting. Intravenous MSC administration shows promise in preclinical and clinical studies, improving outcomes in SCI and other neurological disorders by modulating inflammation and promoting repair. However, optimizing delivery protocols and standardizing treatment parameters remain critical to enhance reproducibility and clinical translation [147–151]. Mendonça et al. [152] investigated intraspinal transplantation of MSC (dose: 1 × 10^7^ cells/mL) in 15 patients with SCI. Neurological assessments post-treatment revealed clinically meaningful symptom alleviation, with universal improvements in tactile sensitivity. Eight patients exhibited enhanced lower limb motor function (notably hip flexor activation), and seven demonstrated sacral sparing, with partial injury classification (AIS grades B/C). Secondary outcomes included improved urologic function in nine patients and detectable changes in somatosensory evoked potentials (SSEPs) in one participant at 3 and 6 months. No severe adverse events were documented, suggesting a favorable safety profile. While the authors posited MSC therapy as a potential modulator of SCI-related dysfunction, they cautioned that confirmation through randomized controlled trials with larger cohorts is imperative to mitigate biases inherent in uncontrolled study designs. Albu et al. [153] demonstrated that intrathecal delivery of WJ-MSCs in SCI patients led to notable improvements in somatosensory function, particularly enhancing pinprick sensation in dermatomes caudal to the injury level. Secondary outcomes revealed therapeutic benefits in neurogenic bladder dysfunction, including increased maximum bladder capacity, mitigation of neurogenic detrusor overactivity, and reduced dyssynergia of the external urethral sphincter. These findings suggest that WJ-MSC therapy may modulate both sensory and autonomic dysfunction post-SCI, though further mechanistic studies are warranted to elucidate the underlying pathways driving these functional recoveries. A randomized, double-blind, placebo-controlled phase II trial evaluated intrathecal UC-MSCs (2××10^6^ cells/kg) in 40 chronic SCI patients. At 12-month follow-up, the UC-MSC group showed significant motor function recovery, enhanced electrophysiological activity (motor-evoked potentials), and reduced lesion size on MRI compared with placebo. Safety data indicated transient headaches/mild meningismus in 25% of recipients, with no severe adverse events. Authors highlighted UC-MSCs’ neurorestorative potential but emphasized the need for phase III trials to confirm long-term efficacy and safety [154].

One effective strategy in MSC-therapy is combination therapy that uses other cell therapy sources. This approach works synergistically by harnessing the immunomodulatory, anti-inflammatory, and neurotrophic effects of MSCs, which are facilitated through their secretome [155]. Recently, Akhlaghpasand et al. [156] conducted a phase II trial to study the effects of intrathecal administration of BM-MSCs and Schwann cells in patients with SCI-induced neurogenic bladder. Results of the study demonstrated that this combined cell therapy could remarkably improve the quality of life and reduce the number of urinary incontinence episodes in patients. A study evaluated the safety and feasibility of co-transplanting autologous olfactory ensheathing cells (OECs) and BM-MSC in patients with chronic, complete SCI classified as AIS grade A. Three individuals with traumatic thoracic-level SCI received an intrathecal injection of a 1:1 mixture of OECs and MSCs, totaling 30 × 10⁶ cells each. Over two years, no serious adverse events were reported, and MRI results showed no abnormal tissue growth. One participant demonstrated partial neurological improvement with an AIS upgrade from A to B and some SCIM III score gains, while the other two showed little to no improvement. Overall, the study indicates that co-transplantation of OECs and MSCs is safe but has limited therapeutic efficacy, suggesting further research is needed [157].

Awidi et al. [158] conducted a trial to evaluate the safety and efficacy of MSC therapy in patients with chronic SCI. The study compared two methods: Group A, intraoperative perilesional administration of 1 × 10^8^ autologous BM-MSCs with monthly (for 3 months) intrathecal injections versus Group B, monthly intrathecal administration of 1 × 10^8^ allogeneic UC-MSCs. While both groups showed improvements, autologous BM-MSCs demonstrated greater motor recovery. The study suggests that both MSC therapies are safe and may improve function in patients with chronic SCI, highlighting the need for larger trials to confirm these initial findings and assess long-term efficacy. Furthermore, a recent study assessed the safety and initial effectiveness of allogeneic WJ-MSCs in patients with chronic complete SCI via three delivery routes. In this study, patients with SCI were administered MSC injections through intrathecal, intravenous, and intramuscular routes. Significant advancements in motor and sensory skills, along with decreased spasticity and improved quality of life, were observed following WJ-MSC treatment. The administration of a target dose of 1 × 10 ^6^ cells/kg was found to be safe across all routes, with only minor complications reported. These included subfebrile fever, headache, and localized muscle pain, all of which resolved within 24 h [159].

Bydon et al. [160] have shown that intrathecal administration of 1 × 10^8^ autologous AD-MSCs improved sensory (80%) and motor function (70%) in patients with SCI. Also, no serious adverse effects were documented during and after the AD-MSCs injection. However, Hur et al. conducted a trial to examine the effect of administering three doses of AD-MSCs (9 × 10^7^ cells in total) in 14 SCI patients. Based on their evidence, AD-MSCs enhanced motor function (36%) and sensory function (71%). The findings indicate that, although the overall cell count remains comparable, the method of administration is vital. This suggests that a single higher dose may yield more significant therapeutic benefits of AD-MSCs in SCI group compared to multiple lower doses.

In general, MSC-based treatments for SCI show a consistent safety profile and promising signs of improvement in motor, sensory, and autonomic function, especially when given intrathecally or intraspinally. When compared with intravenous delivery, direct CNS-targeted approaches appear to yield more measurable functional and electrophysiological benefits, likely because they improve local bioavailability. Both autologous and allogeneic sources (BM-, UC-, WJ-, and AD-MSCs) have shown potential benefits, though comparative superiority remains unclear due to heterogeneity in dosing, timing, and injury chronicity. Combination approaches with Schwann cells or olfactory ensheathing cells suggest possible synergistic effects but have yet to demonstrate robust efficacy. Most trials are still small and have different designs, doses, and cell sources, even though phase I/II results are promising, such as improvements in MRI and neurophysiology. So, SCI is one of the more advanced uses of MSC therapy in terms of translation, but we need large, controlled phase III trials to prove that it works and to identify the best ways to deliver it.

### Cerebral palsy

Cerebral palsy (CP) is a condition characterized by issues with posture and movement resulting from non-progressive injury during brain development. Individuals with CP often experience sensory and perceptual challenges, cognitive difficulties, and behavioral issues and may also develop secondary musculoskeletal problems and epilepsy [161, 162]. Despite advances in obstetrics and perinatology, the occurrence of CP remains at 2 to 3 cases per 1,000 live births. CP is recognized as a leading cause of childhood disabilities and fatalities. In children, CP presents a significant public health challenge that profoundly impacts the quality of life for affected individuals while also placing a financial strain on families and national healthcare systems [163, 164]. Currently, the primary approach to treatment involves orthopedic surgery, hyperbaric oxygen therapy, and neurotrophic medications. However, the clinical effectiveness of these methods remains constrained, as they do not provide significant benefits for CP. Consequently, it is essential for healthcare providers to explore innovative therapeutic alternatives for CP to enhance patients’ quality of life and physical functioning.

Research on stem cell therapy for cerebral palsy is revealing new treatment strategies. Currently, the stem cells primarily used to treat cerebral palsy are MSCs. Clinical studies investigating UC-MSC therapy for CP have demonstrated encouraging results. In a controlled study, 54 patients underwent standard rehabilitation, with 27 receiving four intravenous infusions of UC-MSCs (5 × 10⁷ cells per infusion) alongside rehabilitation, while the control group received only rehabilitation with saline infusions. Functional assessments, including GMFM-88 and CFA scores, revealed significantly greater improvements in the UC-MSC group at 3, 6, 12, and 24 months post-treatment. EEG analysis showed reduced diffuse slow waves in patients with baseline abnormalities, suggesting potential neurophysiological benefits. However, routine MRI revealed minimal structural brain changes. Importantly, no serious adverse events were reported, supporting the safety of this therapeutic approach [165]. A recent randomized controlled trial investigated the safety and effectiveness of UC-MSC transplantation combined with standard rehabilitation for children with CP. Participants received either UC-MSCs or a placebo, with follow-up assessments over 12 months. The study found no significant differences in adverse events between the groups, but patients receiving UC-MSCs showed notable improvements in activities of daily living and motor function. Additionally, imaging results suggested enhanced cerebral metabolic activity in some treated patients. The findings indicate that UC-MSC transplantation could be a promising adjunct to rehabilitation for improving motor and overall functioning in children with CP while highlighting key considerations for future clinical applications. The study’s limitations include a small sample size that hindered subgroup analyses by age and neuroimaging, limiting insights into individual treatment responses. Additionally, insufficient data on cerebral metabolic activity and a lack of inflammatory cytokine measurements restricted comparisons between groups and understanding of underlying mechanisms [166]. The primary mechanism of action of MSCs is believed to stem from their immunomodulatory effects on both humoral and cell-mediated immune responses. This includes inhibiting the proliferation of B cells, T cells, NK cells, dendritic cells, and microglia, as well as decreasing pro-inflammatory cytokine production and blocking neutrophil recruitment [167–170]. In a separate study, Sun et al. [171] assessed the safety and motor function outcomes following treatment with allogeneic umbilical cord blood (UCB) or UC-MSCs in children diagnosed with CP. Ninety-one children aged between 2 and 5 years with CP were assigned randomly to one of three groups: one group was given a high-dose infusion of UCB-MSCs at the start, another group received three doses of UC-MSCs at the beginning, 3 months later, and 6 months later, while the control group followed natural history, receiving UCB-MSCs only at the 12-month mark. The treatments were generally well accepted, with minor infusion reactions and no significant safety issues. After 12 months, both treatment groups exhibited greater improvements in GMFM-66 scores than the natural history group, with the UCB-MSCs group showing a statistically significant improvement. These results indicate that high-dose UCB-MSCs, in particular, might represent a potentially advantageous cell therapy for CP. There are several limitations: families were not blinded to treatment assignment; about 25% of participants missed their 12-month motor evaluations due to COVID-19 (although multiple imputation analyses suggest this did not bias results); multiple CP etiologies were included; and the sample size was insufficient for robust intergroup comparisons.

Boyalı et al. conducted a pilot study to explore the efficacy and safety of UC-MSC in CP patients who were unable to stand or walk without assistance. Four patients received six treatments of allogeneic UC-MSCs at a dose of 1 × 10^6^ cells/kg, administered via intrathecal, intravenous, and intramuscular routes. Following treatment, improvements in spasticity were observed at 2, 4, and 12 months, and enhancements in gross motor and manual abilities were noted at 12 months, while overall motor and cognitive functions remained largely unchanged. However, the study had limitations, including a small sample size, a wide age range among participants, and the absence of a control group. Despite these limitations, the findings contribute valuable insights into the potential of UC-MSC therapy for CP and warrant further investigation in larger, controlled trials [172].

Overall, clinical studies investigating MSC therapy for cerebral palsy have reported encouraging safety outcomes along with modest improvements in motor function, particularly when treatment is combined with rehabilitation programs. Most trials have used UC-MSCs or UCB-MSCs delivered intravenously, and several have shown improvements in measures such as motor function and activities of daily living. However, neuroimaging findings have generally shown only minimal structural changes in the brain. This observation suggests that the therapeutic benefits of MSCs may primarily result from their immunomodulatory and neurotrophic effects rather than from direct tissue regeneration. In addition, variations in cell sources, dosing regimens, and patient characteristics, together with relatively small sample sizes, make direct comparisons between studies difficult. Although MSC therapy appears promising as an adjunct to conventional rehabilitation for children with CP, larger, well-designed clinical trials are still necessary to determine its long-term efficacy and establish optimal treatment protocols.

### Parkinson’s disease

PD is a multifaceted disorder associated with aging that predominantly impacts people older than 65 years. It is marked by the deterioration of dopaminergic neurons within the substantia nigra, resulting in both motor and non-motor impairments due to reduced dopamine levels in the striatum [173, 174]. PD ranks as the second most common neurodegenerative disorder following Alzheimer’s disease and exhibits the fastest growth rate among all neurological conditions [175]. The key pathological features of PD include the gradual and ongoing degeneration of dopaminergic neurons in the nigrostriatal pathway and the substantia nigra pars compacta [176]. Additionally, the presence of neuronal α-synuclein aggregates known as Lewy bodies and neuroinflammation in different areas of the brain is also significant [177, 178]. In the pathology of PD, activation of the NF-κB pathway in microglia stimulates the secretion of inflammatory chemokines (such as RANTES and eotaxin), which increases CD8 + T cell infiltration into the substantia nigra and enhances cytotoxic activity against dopaminergic neurons. Furthermore, microglia internalize α-Synuclein and present it to CD4 + and CD8 + T cells as an antigen, thereby initiating autoimmune reactions. These interconnected processes ultimately result in the apoptosis of dopaminergic neurons [179, 180]. The degeneration of dopamine-producing neurons in the substantia nigra pars compacta leads to the characteristic symptoms of PD, categorizing it as a movement disorder marked by resting tremor, postural instability, rigidity, and bradykinesia [181, 182]. Pharmacological approaches can enhance dopamine activity and alleviate the motor symptoms of PD, particularly in its initial phases. Nonetheless, while these treatments can be beneficial, they do not address, and may even worsen, the non-motor symptoms of PD, including postural hypotension and neuropsychiatric issues [183–185].

The findings of the studies indicate that MSCs can speed up the removal and breakdown of α-Syn through various multitarget modifications, including the regulation of microglia activation, the autophagic pathway, endocytosis, and the secretion of proteases [186–188]. A study showed that administering MSCs via cerebral arteries in patients with PD resulted in favorable outcomes for 17 participants: all individuals in the treatment group survived, and their motor function assessment scores remained stable for at least 6 months during the 12-month follow-up. One individual passed away 9 months post-injection due to causes unrelated to the cell infusion or the progression of the disease [189]. A recent clinical study by Lemus et al. analyzed the secondary outcomes in PD patients and demonstrated that three repeated infusions of allogenic BM-MSCs could result in the improvement of eGFR and serum creatinine levels in patients with PD. This signifies a crucial initial step in demonstrating the safety and preliminary efficacy of allogenic BM-MSCs as a potential treatment to maintain and possibly enhance secondary outcomes in PD patients [190].

Another study evaluated the safety and potential benefits of autologous BM-MSC transplantation in PD. Seven PD patients received a single unilateral dose of BM-MSCs, which were stereotaxically transplanted into the sublateral ventricular zone, and were subsequently monitored over an extended follow-up period. Baseline motor function was assessed using standard clinical scales, and during follow-up, some patients demonstrated improvements in their motor symptoms, including enhanced performance on global rating scales and subjective improvements in facial expression, gait, and reduced episodes of freezing. Additionally, a few patients were able to lower their PD medication dosages. Although no serious adverse events were reported, the study’s small sample size and uncontrolled design limit definitive conclusions regarding treatment efficacy. These preliminary findings support the safety of BM-MSC transplantation in PD and highlight the need for larger, controlled studies to further evaluate its therapeutic potential [191].

Transforming Growth Factor-beta 1 (TGF-β1) is a pivotal cytokine that is essential for both innate and adaptive immunity. It is instrumental in facilitating neurogenesis and gliosis, as well as in the regulation of neural patterning, myelination, and the development of microglia throughout brain maturation [192, 193]. TGF-β1 plays a crucial role in the development and maintenance of microglial cells. The absence of TGF-β1 leads to the downregulation of genes such as tmem119 and p2ry12, which are essential for microglial homeostasis. Conversely, this deficiency results in the upregulation of genes associated with cellular injury, including Axl and APOE [194]. In parallel with this, a recent study has explored the therapeutic potential of clinical-grade, hypoxia-preconditioned olfactory mucosa-derived MSCs (hOM-MSCs) for PD. In both PD models and a cohort of severe PD patients, hOM-MSC treatment improved neural function and modulated the neuroinflammatory response. They identified TGF-β1 as a key factor secreted by hOM-MSCs that enhances mitochondrial function in dopaminergic neurons by promoting microglial regulation and autophagy homeostasis via the ALK/PI3K/Akt signaling pathway. Furthermore, intraspinal transplantation of hOM-MSCs in PD patients not only improved neurological function but also did so without adverse effects. These findings underscore the promise of hOM-MSCs and potentially TGF-β1 as a standalone or adjunct therapy as a novel neuroprotective strategy for PD, meriting further clinical investigation [195].

Overall, studies investigating MSC-based therapies for PD suggest a favorable safety profile and potential neuroprotective effects, although the clinical evidence remains limited. Early clinical studies using different MSC sources and delivery routes, including intra-arterial and stereotactic transplantation, have reported stabilization or modest improvements in motor symptoms. These benefits are thought to arise mainly from the immunomodulatory and neuroprotective properties of MSCs, including reducing neuroinflammation, promoting the clearance of α-synuclein aggregates, and secreting trophic factors such as TGF-β1. However, most studies involve small patient cohorts and lack rigorous control groups, making it difficult to draw firm conclusions about long-term efficacy. Therefore, larger and well-designed clinical trials are still needed to determine the optimal cell sources, delivery strategies, and therapeutic potential of MSCs in PD.

### Ischemic stroke

Ischemic stroke is a medical condition resulting from a disruption in blood circulation within the brain, which causes the death of nerve cells or the softening of brain tissue. The loss of a significant number of these terminally differentiated cells results in permanent damage to the brain. Prompt restoration of blood flow in the affected area and minimizing neuronal cell death are critical aspects of treating ischemic stroke. Nonetheless, therapies like thrombolysis and mechanical thrombectomy are advantageous for only 5% of patients due to limited therapeutic windows and serious treatment complications [196, 197]. Presently, thrombolysis and mechanical thrombectomy have significantly advanced the management of ischemic stroke. However, these methods are not without their challenges, including restricted time frames for intervention, risks associated with hemorrhage, issues related to availability, and the potential for treatment to be ineffective [196, 198].

**Fig. 3:**
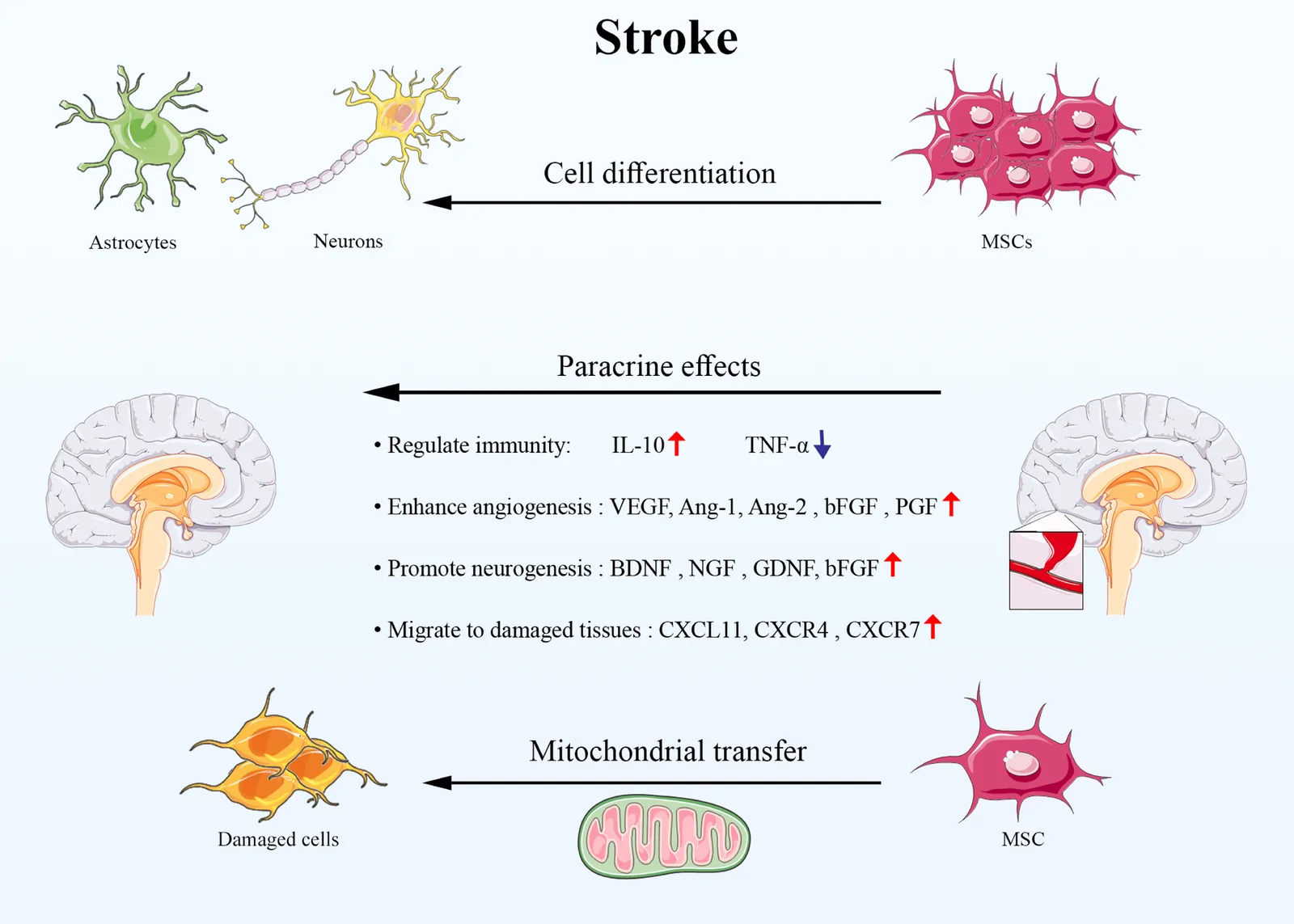
Mechanisms of MSC therapy for the treatment of stroke. MSCs promote neurological recovery after stroke through multiple complementary mechanisms, including differentiation into neural lineage cells, paracrine secretion of immunomodulatory, angiogenic, and neurotrophic factors that suppress inflammation, enhance angiogenesis and neurogenesis, and facilitate homing to injured brain tissue, as well as mitochondrial transfer to damaged cells to restore cellular bioenergetics and improve cell survival

Increasing evidence suggests that MSCs influence the pathological mechanisms of ischemic stroke (Fig. 3) through various targets and temporal phases, including the reduction of inflammation, modulation of immune function, inhibition of apoptosis, and promotion of neurovascular, white matter, and synaptic remodeling during the acute, subacute, and chronic stages of ischemic stroke [199]. A phase II clinical trial employing a prospective, randomized, controlled, observer-blinded design was conducted to assess the clinical safety, feasibility, and mechanistic underpinnings of allogenic BM-MSCs administered through intrathecal infusion in individuals with cerebral infarction localized to the middle cerebral artery territory and presenting NIHSS scores of 15–25. Based on sample size estimation, 118 participants diagnosed with ischemic stroke 30 to 90 days post-onset were randomized into experimental (*n* = 59) and control (*n* = 59) cohorts. Eligible participants in the experimental group received four weekly intrathecal administrations of allogenic BM-MSCs at a dosage of 1 × 10⁶ cells per infusion. The primary endpoint assessed was the modified Rankin Scale score at 90 days post-intervention, while secondary endpoints encompassed metrics evaluating safety profiles and procedural feasibility [200]. Jaillard et al. conducted an RCT (ISIS-HERMES) to evaluate the safety, feasibility, and efficacy of intravenous autologous BM-MSCs in subacute stroke patients. In this study, 118 patients aged 18–70 years who experienced a moderate-to-severe ischemic carotid stroke within two weeks were randomized in a 2:1 ratio to receive either MSCs or standard care. Over a two-year follow-up, the treatment was found to be safe and feasible. Although global outcome measures showed no significant differences, improvements were noted in motor function assessments and task-related activity in the primary motor cortex. These findings suggest that intravenous MSC therapy may enhance motor recovery in stroke patients through mechanisms of sensorimotor neuroplasticity [201]. In a different study, Ruiz et al. [202] performed a phase IIa, randomized, double-blind, placebo-controlled pilot trial to assess the safety and effectiveness of intravenous AD-MSCs in patients who experienced moderate to severe ischemic stroke. A total of 13 patients (4 who received AD-MSCs and 9 who received placebo) finished the study, which included a follow-up period of 24 months. The results indicated that the administration of AD-MSCs was safe, with no adverse events or tumor formation related to the injections. Although there was a tendency for the AD-MSC group to have lower NIHSS scores at the 24-month mark, no significant differences were found in modified Rankin Scale scores between the two groups. These results suggest that intravenous AD-MSC treatment is safe during the early post-stroke phase, but further studies with larger populations are necessary to evaluate its therapeutic effectiveness. The primary constraint of the study is its limited sample size, which arises from its pilot clinical trial design. Additionally, the small number of patients in the treatment group was affected by technical difficulties encountered during the manufacturing process, along with the introduction of exclusion criteria after randomization. In a study conducted by Lee and colleagues in 2022, 54 patients with ischemic stroke were administered intravenous injections of autologous BM-MSCs. The trial results demonstrated that motor function improvement, as measured by the Fugl-Meyer assessment score, was notably greater in the MSC group than in the control group. Neuroimaging showed that the fractional anisotropy of the corticospinal tract and the posterior limb of the internal capsule did not decline in the MSC group; however, it significantly reduced in the control group at 90 days post-randomization. There was a significant increase in interhemispheric connectivity and ipsilateral connectivity in the MSC group. The change in interhemispheric connectivity indicated a significant difference between the two groups [203].

UC-MSCs come from younger tissue, giving them stronger self-renewal and differentiation capacity than MSCs from older sources like bone marrow. They are also less likely to trigger immune rejection, making them a promising option for allogeneic cell therapies [204]. In a Phase I trial, Chan et al. revealed that UC-MSCs (UMC119-06) were generally well-tolerated by patients with acute ischemic stroke, with no dose-limiting toxicities observed. While the safety evaluation indicated a significant occurrence of serious adverse events in both dose cohorts, none were determined to be directly attributable to UMC119-06. Importantly, no significant immunogenic reactions, thromboembolic events, or organ dysfunctions directly linked to the administration of UMC119-06 were observed. Moreover, the decrease in infarct volume observed on brain MRI supports the potential neuroprotective and reparative benefits of UC-MSCs. These encouraging trends seen in clinical outcomes imply that UC-MSCs could contribute to improving recovery and fostering functional independence in patients with acute ischemic stroke [205]. Recently, a study compared two routes of administration (intrathecal versus intravenous) of UC-MSCs in 32 patients. Their research showed that UC-MSC therapy, given through either the intravenous or intrathecal method, resulted in notable enhancements in individuals with ischemic stroke during the subacute and chronic phases. These enhancements were noted in neurological functions, motor skills, muscle tone, and overall quality of life. The researchers hypothesized that intrathecal cell therapy would yield superior outcomes compared with intravenous administration; however, the study’s findings did not corroborate this assumption [206].

Taken together, current clinical evidence indicates that MSC-based interventions for ischemic stroke are generally well tolerated and technically feasible, with several studies reporting improvements in motor performance and indicators of neural network reorganization. Therapeutic effects have been observed following both intravenous and intrathecal delivery, although comparative studies have not consistently demonstrated superiority of either route. The available data suggest that MSCs may facilitate recovery primarily by modulating post-stroke inflammation and promoting neurovascular remodeling rather than by directly replacing lost neurons.

### Alzheimer’s disease

Alzheimer’s disease is among the most common forms of dementia, impacting approximately 50 million people worldwide. Given that age is a significant factor in its development and considering the rapid aging of the population [207]. Dementia is characterized by memory impairment, confusion, cognitive deterioration, difficulty managing everyday activities, and changes in behavior [208]. Alzheimer’s disease is marked by two key pathological features: amyloid plaques (Aβ) and neurofibrillary tangles (NFTs). The Aβ pathology originates from the incorrect cleavage of the amyloid precursor protein (APP), which leads to the formation of Aβ monomers that cluster together, forming oligomeric Aβ and ultimately leading to the aggregation into Aβ fibrils and plaques [209, 210]. NFTs result from the accumulation of hyperphosphorylated Tau protein, a protein associated with microtubules that helps maintain their stability [211, 212]. In Alzheimer’s disease, tau protein undergoes phosphorylation at numerous sites, causing the detachment of microtubules from the axon and disrupting intracellular transport. Additionally, hyper-phosphorylated tau aggregates into NFTs, which impair overall cellular function and ultimately result in neuronal death [213, 214]. The primary obstacle is creating successful disease-modifying therapies that can halt or reduce the advancement of Alzheimer’s. Scientists are diligently exploring different strategies to tackle this issue and offer improved solutions for patients down the line, one of which involves the use of MSCs.

Kim et al. conducted a phase I clinical trial to assess the safety and tolerability of repeated intracerebroventricular administration of UC-MSCs in patients with Alzheimer’s disease. In this study, nine patients underwent implantation of an Ommaya reservoir into the right lateral ventricle four weeks prior to treatment and were subsequently administered three MSC injections at four-week intervals. The trial compared two dosing regimens, with three patients receiving a low dose (1.0 × 10^7^ cells/2 mL) and six receiving a high dose (3.0 × 10^7^ cells/2 mL). During a 36-month extended follow-up in five participants, no additional serious adverse events were reported. This study showed that UC-MSC injections produced promising short-term effects, with immediate reductions in CSF total tau, phosphorylated tau, and Aβ42, and a spike in inflammatory markers. However, these effects were transient, as the biomarker levels returned to baseline by four weeks, possibly due to the short lifespan of the MSCs or dilution effects from post-injection saline. These results suggest that more frequent dosing may be required, a strategy that is now being further investigated in a randomized phase IIa trial. The study’s limitation is that the UC-MSC injection dose was based on mouse studies, using a dosage ratio related to CSF volumes between mice and humans. This method may not accurately represent the optimal dose for Alzheimer’s patients. Further detailed studies are needed to establish the most effective cell dose for humans [215].

**Table 1:** Clinical studies of MSC therapy in neurological disorders

Neurological disorder	Study reference	MSC source	Dosage	Administration route	Key findings	Limitations
Multiple Sclerosis (MS)	Jamali et al. [103]	UC-MSCs	2 doses of 150 × 10⁶ cells	Intrathecal	Improved disability scores, reduced lesion load, enhanced cortical thickness, and down-regulated TNF-α and TAP-1	Small cohort; requires larger trials
	Harris et al. [104]	BM-MSCs	9.4 × 10⁶ cells (mean)	Intrathecal	Improved EDSS and T25FW; reduced CSF CCL2	No placebo control; unblinded
	Harris et al. [105]	BM-MSCs	1 × 10⁷ cells/dose (6 doses)	Intrathecal	Improved bladder function, reduced rate of grey matter atrophy, increased MMP9 and decreased CCL2 levels	No overall efficacy; subgroup analysis only
	Shokati et al. [110]	placenta-MSCs	3 × 10⁶ cells/kg	Intravenous	Reduced dysfunction, MS symptoms, systemic inflammation and improved cognition	Small sample, short follow-up, and lack of control groups
	Petrou et al. [111]	BM-MSCs	1 × 10⁶ cells/kg	Intrathecal/Intravenous	Improved EDSS and MRI outcomes; intrathecal superior to intravenous	Requires phase III trials
	Petrou et al. [113]	BM-MSCs	1 × 10⁶ cells/kg	Intrathecal/Intravenous	Reduced CSF NF-L and CXCL13 (neuroprotection)	Biomarker variability; Statistical analysis
	Petrou et al. [114]	BM-MSCs (MSC-NG01)	1 × 10⁶ cells/kg	Intrathecal	Reduced NFL and GFAP, downregulated CNS inflammation	Statistical analysis, small sample and the open-label design
	Phase II [115]	BM-MSCs	10–12.5 × 10⁷ cells (3 doses)	Intrathecal	Improved T25FW/9-HPT (19% of patients); increased neuroprotective CSF factors	Open-label; no placebo; limited biomarker analysis
	Uccelli et al. [117]	BM-MSCs	Not specified	Intravenous	Safe but no reduction in gadolinium-enhancing lesions	Ineffective for active inflammation
Amyotrophic Lateral Sclerosis (ALS)	Syková et al. [130]	BM-MSCs	15 ± 4.5 × 10⁶ cells	Intrathecal	Slowed ALSFRS decline; stable FVC	Small sample; no control
	Barczewska et al. [129]	WJ-MSCs	30 × 10⁶ cells (3 doses)	Intrathecal	Improved ALSFRS scores	No control group
	Staff et al. [131]	AD-MSCs	1 × 10^7^ 5 × 10^7^ 1 × 10^8^	Intrathecal	No symptom improvement; disease progression	Adverse CSF changes
	Karimi et al. [132]	WJ-MSCs	60 × 10⁶ cells/kg	Intrathecal/Intravenous	Reduced muscle tremors and fasciculations, increased muscle strength, improved salivation, swallowing, and speech	Small sample size, no control group
	Siwek et al. [133]	BM-MSCs	10 × 10⁶ cells (3 doses)	Intrathecal	Modest slowing in rapid ALS progression	Small subgroup; autologous challenges
	Alkhazaali-Ali et al. [136]	BM-MSCs	1 × 10⁶ cells/kg	Intrathecal/Intravenous	Reduced TNF-α levels in CSF, Upregulation IL-10, and stabilized NFL levels	Small sample size, no control group, short follow up period
	Siwek et al. [139]	BM-MSCs	1 × 10⁶ cells/kg	Intrathecal	CSF proteomic changes (ECM remodeling)	High dropout rate; no controls
Spinal Cord Injury (SCI)	Mendonça et al. [152]	BM-MSCs	1 × 10⁷ cells/mL	Intraspinal	Improved motor/sensory function; safe	Uncontrolled; small cohort
	Albu et al. [153]	WJ-MSCs	1 × 10^6^ cells	Intrathecal	Enhanced somatosensory function; improved bladder control	Mechanistic uncertainty
	Phase II Trial [154]	UC-MSCs	2 × 10⁶ cells/kg	Intrathecal	Motor recovery; reduced lesion size	Transient headaches
	Akhlaghpasand et al. [156]	BM-MSCs	5 × 10^6^ cells per mL in 6 mL saline	Intrathecal	Improved bladder and urinary tract dysfunction	No control group, short follow up period
	Co-transplant [157]	BM-MSCs + OECs	30 × 10⁶ cells each	Intrathecal	Safe but limited functional improvement	Small sample; incomplete SCI types
	Awidi et al. [158]	BM-MSCs/UC-MSCs	1 × 10⁸ cells	Perilesional/Intrathecal	Showed superior motor recovery	Open-label; longer follow-up needed
	Kaplan et al. [159]	WJ-MSCs	1 × 10⁶ cells/kg	Intrathecal, Intravenous, Intramuscular	Improved FIM Motor scores, reduced spasticity	No control group
	Bydon et al. [160]	AD-MSCs	100 × 10^6^ cells	Intrathecal	Improved sensory and motor	No control group
Cerebral Palsy (CP)	Huang et al. [165]	UC-MSCs	5 × 10⁷ cells (4 doses)	Intravenous	Improved GMFM-88/CFA scores; reduced EEG abnormalities	Limited MRI changes
	Gu et al. [166]	UC-MSCs	Not specified	Intravenous	Improved motor function; enhanced cerebral metabolism	Small sample; no cytokine analysis
	Sun et al. [171]	UC /UCB-MSCs	High-dose AlloCB/hCT-MSC	Intravenous	Improved GMFM-66 scores	Unblinded; COVID-19 disruptions
	Boyalı et al. [172]	UC-MSCs	1 × 10⁶ cells/kg (6 doses)	Intrathecal/IV/IM	Improved spasticity; no cognitive changes	Small sample; no control
Parkinson’s Disease (PD)	Canesi et al. [189]	BM-MSCs	1.7 (1.2–2.0) × 10^6^ cells/kg	Cerebral arteries	Stable motor function for 6 + months	Unclear MSC source/dose
	Venkataramana et al. [191]	BM-MSCs	1 × 10^6^ cells/kg	Sublateral ventricular	Improved motor function; reduced PD medication dosages	Small sample; uncontrolled
	Zhou [195]	hOM-MSCs	5 × 10^7^ cells	Intraspinal	Improved neurological function; TGF-β1 secretion	Requires phase III validation
	Martinez-Lemus [190]	Allo-BM-MSCs	10 × 10^6^ cells/kg	Intravenous	Improved eGFR Reduced serum creatinine	No healthy controls
Stroke	Jaillard et al. [201]	BM-MSCs	1 × 10⁶ cells (4 doses)	Intrathecal	Improved motor function; neuroplasticity	Global outcomes unchanged
	Ruiz et al. [202]	AD-MSCs	1 × 10^6^ cells/kg	Intravenous	Safe; reduced NIHSS scores	Small sample; technical challenges
	Lee et al. [203]	BM-MSCs	Not specified	Intravenous	Preserved corticospinal integrity; enhanced connectivity	No control group
	Nguyen et al. [206]	UC-MSCs	1.5 × 10^6^ cells/kg	Intrathecal Intravenous	Improved neurological function, motor ability, muscle tone, and quality of life	Small sample size
	Chan et al. [205]	UC-MSCs (UMC119-06)	1 × 10^6^ cells/kg 5 × 10^6^ cells/kg	Intravenous	Reduced infarct volume, Enhanced VEGF and HGF, neuroprotection, and secretion of regenerative paracrine factors	Small sample size No control group
Alzheimer’s Disease (AD)	Kim et al. [215]	UC-MSCs	1–3 × 10⁷ cells (3 doses)	Intracerebroventricular	Transient CSF biomarker improvements	Dosing based on animal models; short-lived effects

## Clinical challenges of MSCs in neurological diseases

The therapeutic application of MSCs in neurological diseases faces significant challenges that span biological, technical, and clinical domains. Mechanistic uncertainties remain a major hurdle, as the immunosuppressive and regenerative effects of MSCs depend on complex, context-dependent interactions with inflammatory microenvironments. While MSCs modulate immune cells and secrete trophic factors, the precise molecular pathways driving these effects are not fully understood, leading to variable outcomes in diseases like MS and ALS [40–42, 46].

### MSC product heterogeneity

Heterogeneity in MSC sources and preparation further complicates clinical translation, as MSCs derived from BM, UC, or AD exhibit differences in proliferation, differentiation potential, and secretory profiles. For instance, UC-MSCs demonstrated superior motor recovery in SCI compared to BM-MSCs, while AD-MSCs failed to improve ALS symptoms, highlighting the impact of tissue origin on therapeutic efficacy [131, 154, 158]. It has become increasingly evident that the minimal criteria proposed by the ISCT are insufficient to fully define MSC identity, as substantial heterogeneity exists at multiple biological levels. First, MSCs derived from different donors exhibit considerable functional variability, influenced by factors such as age, health status, and other individual-specific characteristics. Second, MSCs isolated from distinct tissue sources, including adipose tissue and bone marrow, differ in their phenotypic profiles, surface marker expression, and differentiation potential. These observations indicate that MSCs represent a heterogeneous cell population rather than a uniform therapeutic entity [216].

To better delineate functionally distinct subpopulations within heterogeneous MSC preparations, considerable efforts have been directed toward identifying characteristic cell surface markers and molecular signatures. Studies have shown that single-cell-derived colonies composed of rapidly proliferating cells possess markedly enhanced colony-forming capacity, suggesting the existence of highly clonogenic MSC subsets. Several surface markers, including STRO-1, CD146, and CD271, have been associated with these highly proliferative populations and are increasingly recognized as potential indicators of MSC subsets with superior self-renewal and regenerative potential [217].However, the presence of similar surface marker profiles does not necessarily predict functional equivalence among MSC subpopulations. Studies have demonstrated that cell subsets expressing comparable phenotypic markers can exhibit markedly different chondrogenic differentiation capacities, even when cultured under identical experimental conditions. These findings suggest that conventional surface markers alone are insufficient to fully capture the functional diversity of MSCs and underscore the need for more comprehensive molecular and functional characterization of distinct MSC subsets [218].

Advances in transcriptomic profiling, particularly RNA sequencing and microarray analyses, have identified gene expression signatures that can predict the lineage commitment and differentiation potential of MSCs. Among the key regulators of osteogenic differentiation are the transcription factors Osterix (SP7) and distal-less homeobox 5 (DLX5), both of which play pivotal roles in osteoblast development and bone formation. In contrast, adipogenic differentiation is primarily governed by peroxisome proliferator-activated receptor gamma (PPAR-γ) and CCAAT/enhancer-binding protein alpha (C/EBPα), which orchestrate the transcriptional programs required for adipocyte specification and maturation. These findings highlight the value of transcriptional profiling for identifying functionally distinct MSC subpopulations and predicting their differentiation trajectories [219].

### Route of administration and biodistribution

Optimization of delivery protocols is another critical challenge, as route, timing, and dosage influence outcomes. Intrathecal administration in MS and ALS enhanced immunomodulation but carried risks of arachnoiditis and neuropathic pain, whereas intravenous delivery in stroke improved motor recovery but faced issues with cell homing [103, 115, 130, 200, 201, 203]. Dose-dependent effects were evident in MS, where two UC-MSC doses outperformed a single dose, yet optimal regimens remain undefined for most conditions [103].

Clinical trial design limitations, including small sample sizes, lack of placebo controls, and short follow-up periods, undermine the reliability of results. Phase I/II trials in PD and ALS involved fewer than 20 participants, limiting statistical power, while open-label designs in CP and SCI introduced bias [130, 133, 165, 191, 220]. Patient heterogeneity, such as varying baseline disability in MS or progression rates in ALS, further complicates outcome interpretation [27, 46]. Engraftment and survival challenges also persist, as MSCs exhibit poor long-term retention in hostile CNS microenvironments. Transient biomarker improvements in Alzheimer’s disease and inconsistent neuroimaging changes in CP suggest limited MSC persistence, necessitating repeated dosing or engineered strategies to enhance survival [165, 215, 220]. Standardization and scalability issues arise from variability in isolation protocols, donor age, and culture conditions, which hinder reproducibility. Autologous therapies face logistical hurdles in rapidly progressing diseases like ALS, whereas allogeneic “off-the-shelf” products, though scalable, require rigorous quality control to address immunogenicity concerns [133, 154, 165].

A significant challenge in MSC-based therapy is the limited efficacy of cell homing and distribution after systemic administration. After intravenous injection, a large number of MSCs become trapped in the pulmonary circulation. This is known as the pulmonary first-pass effect and limits the number of cells that reach the target tissue [221]. Furthermore, evidence indicates that cell persistence after administration is minimal; one report noted that less than 5% of transplanted MSCs were identifiable at the injection site within hours of local delivery [222, 223]. These results show that low retention and shorter survival after transplantation greatly reduce the effectiveness of the treatment. Various strategies have been investigated to improve engraftment and functional outcomes. Hypoxic preconditioning has been shown to enhance MSCs’ reparative capacity. Cells that were preconditioned had a higher angiogenic potential and were better able to restore damaged myocardium than cells that were grown in normal oxygen levels [224]. Researchers have also investigated biomaterial-based encapsulation methods to help MSCs remain alive and healthy in vitro, but more research is needed to determine whether they work in vivo. Modifying the host microenvironment may also help cells survive after transplantation. For example, administering antioxidants such as vitamin C may reduce oxidative stress and protect engrafted MSCs from damage. These methods all show how important it is to improve both the properties of the cells themselves and the environment of the tissue that will receive them in order to get the best results from treatment [225]. Additionally, direct administration of homing factors may enhance the efficacy of MSCs’ homing and distribution after systemic administration. Studies have shown that injecting SDF-1 into the ischemic myocardium and ischemic brain lesions can enhance the homing of MSCs [226, 227].

### The inflammatory microenvironment

MSCs are severely restricted in their ability to survive in the hostile CNS microenvironment, even when they effectively reach target tissues. A study was conducted to investigate autopsy materials from patients who had received MSCs mismatched for human leukocyte antigen through transplantation. The results of their research suggested that the long-term presence of these transplanted cells was minimal, primarily due to their inability to survive after intravenous administration or to immune rejection by the recipient [228]. They suggested that MSCs control their function via a “hit and run” approach, instead of needing to be continuously implanted in injured tissues. Despite the fact that MSCs perform therapeutic functions through a brief “hit and run” approach, improving their immune evasion and prolonging their in vivo survival could improve clinical outcomes and decrease the likelihood of patients developing allergies to donor antigens [229].

### Validated biomarkers for predicting MSC therapeutic response

The lack of validated biomarkers to predict therapeutic response or monitor MSC activity impedes personalized approaches. While reductions in CSF neurofilament light chain (NF-L) and CXCL13 correlated with MSC efficacy in MS, and proteomic shifts in ALS hinted at extracellular matrix remodeling, these findings lack mechanistic clarity and require validation [113, 139]. Safety concerns, though generally minimal, include procedural risks such as arachnoiditis in MS and back pain in CP, underscoring the need for long-term monitoring [115, 172]. Theoretical risks of ectopic differentiation or tumorigenicity, though unreported in clinical studies, warrant continued vigilance. Addressing these challenges demands collaborative efforts to standardize MSC production, validate biomarkers, and conduct large, randomized, controlled trials with stratified cohorts. Innovations such as engineered MSCs with enhanced survivability or targeted exosome-based delivery systems may improve therapeutic consistency, paving the way for transformative treatments in neurological diseases [220].

### Replicative senescence and differentiation constraints of MSCs

MSCs possess a finite proliferative lifespan and cannot be expanded indefinitely in vitro. Prolonged culture leads to replicative senescence, characterized by progressive declines in proliferative capacity, self-renewal, and differentiation potential. Studies demonstrated that MSCs could be maintained in culture for an average of approximately 118 days and underwent a mean of eight passages before exhibiting pronounced senescent features. Notably, both population doubling (PD) and telomere length (TL) decreased progressively with serial passaging. By the tenth passage, PD declined markedly, whereas telomere shortening occurred at a rate of nearly 100 base pairs every two passages. These changes were accompanied by impaired osteogenic and adipogenic differentiation, indicating that extended culture expansion compromises MSC stemness and functional competence [230].

Additional investigations have revealed that long-term culture also alters the clonal heterogeneity of MSC populations. With increasing passage number, MSCs display substantial heterogeneity in colony formation and clonal expansion, accompanied by a progressive reduction in colony-forming unit-fibroblasts (CFU-Fs). Indeed, CFU-F activity becomes nearly undetectable after more than 20 passages. Concurrently, highly potent MSC subpopulations with superior differentiation capacity progressively disappear during culture expansion. Collectively, these findings suggest that extensive ex vivo expansion diminishes the regenerative potential of MSC preparations and emphasize the importance of utilizing cells at earlier passages to maximize therapeutic efficacy [231].

Although MSCs are widely recognized for their trilineage differentiation potential, their ability to generate derivatives of all three germ layers remains relatively limited and highly variable. Studies have shown that only a small fraction of MSCs can differentiate into ectodermal derivatives such as neurons [232], while similarly low efficiencies have been reported for differentiation into endodermal lineages, including insulin-producing pancreatic β-like cells [233]. In contrast, osteogenic differentiation, representing mesodermal commitment, is generally more efficient but still occurs in only a subset of the overall cell population [234]. These limitations in lifespan, clonal expansion, and differentiation efficiency have important translational implications because senescent MSC populations exhibit profound phenotypic and functional alterations that are likely to reduce their therapeutic performance following transplantation.

## Strategies to enhance the therapeutic efficacy of MSCs

**Fig. 4:**
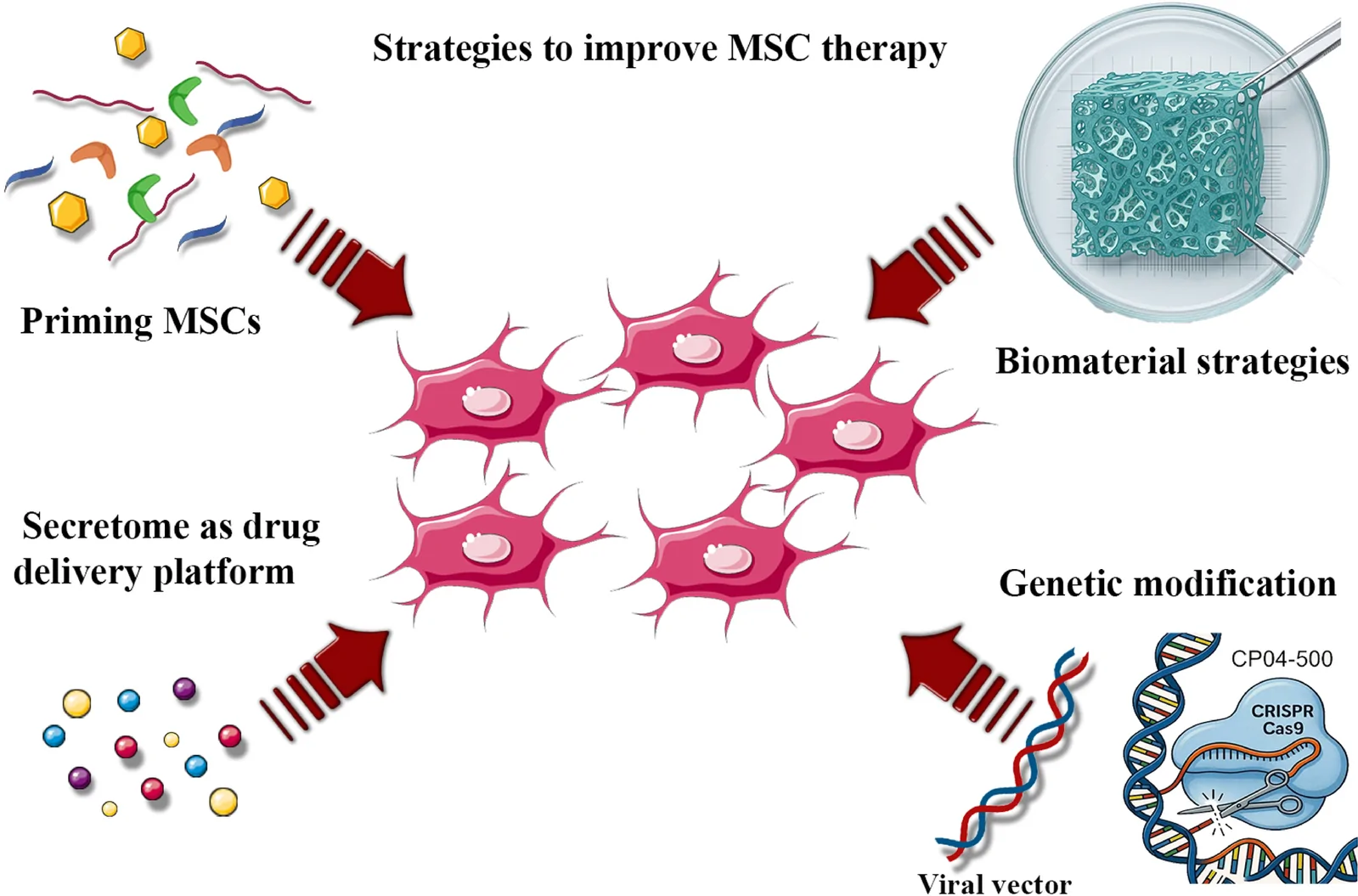
Current strategies to enhance MSC-based therapy. Therapeutic efficacy of MSCs can be improved through several complementary approaches: (1) genetic engineering, including viral vector-mediated gene delivery and CRISPR/Cas9 genome editing, to enhance MSC homing, proliferation, survival, and regenerative potential; (2) MSC priming (preconditioning) using small molecules, hypoxia, or biomaterial-derived mechanical and biochemical cues to improve cell function, persistence, and therapeutic activity; (3) biomaterial-based engineering, which provides supportive scaffolds with optimized dimensionality, stiffness, surface chemistry, topographical features, and microarchitecture to promote MSC survival, engraftment, and function; and (4) therapeutic exploitation of the MSC secretome, including EVs and soluble bioactive factors, as a cell-free drug delivery platform for regenerative medicine

Although MSC therapy has generally demonstrated a favorable safety profile, with no consistent evidence of treatment-related serious adverse events, mild and transient complications such as low-grade fever within the first 24 h and temporary pain at the injection site are relatively common. To further enhance therapeutic efficacy while minimizing potential adverse effects, several strategies have been developed to optimize MSC-based interventions. These approaches can be broadly categorized into four main areas: genetic modification and priming techniques aimed at enhancing the intrinsic biological properties of MSCs, biomaterial-based strategies designed to improve the cellular microenvironment and delivery efficiency, and the therapeutic application of MSC-derived secretome products (Table 2). Collectively, these strategies seek to overcome current limitations of MSC therapy and improve its translational and clinical outcomes (Fig. 4).

### Priming of MSCs

Biochemical and biophysical factors within the local microenvironment play critical roles in regulating the behavior, differentiation capacity, and therapeutic functions of MSCs. Factors such as oxygen tension, inflammatory mediators, extracellular matrix composition, mechanical stimuli, and cell–cell interactions can profoundly influence the survival, secretory activity, and regenerative potential of MSCs. Recognizing the importance of these environmental signals, considerable efforts have been devoted to manipulating the MSC microenvironment to enhance their therapeutic efficacy. By modulating extrinsic conditions before or after transplantation, researchers aim to improve MSC engraftment, functional activity, and overall clinical performance [235, 236]. One of the most widely employed approaches for enhancing MSC function is preconditioning, or priming, with pro-inflammatory mediators such as IFN-γ, TNF-α, IL-1α, and IL-1β. Exposure to these cytokines can substantially augment the immunomodulatory, regenerative, and neuroprotective properties of MSCs, thereby improving their survival and therapeutic efficacy after transplantation. In recent years, an expanding range of priming strategies has been developed to further optimize MSC performance [237, 238]. Based on the nature of the stimuli, these approaches can generally be classified into three major categories: priming with bioactive small molecules, hypoxic preconditioning, and biomaterial-based priming. Collectively, these strategies seek to modulate MSC behavior and enhance their functional competence, providing promising avenues for improving the clinical outcomes of MSC-based therapies.

Several primed MSC products have progressed to clinical evaluation, among which NurOwn, developed by the company Brainstorm Cell Therapeutics, represents one of the most extensively investigated examples. NurOwn is generated by inducing MSCs to secrete elevated levels of multiple neurotrophic factors, including GDNF, BDNF, VEGF, and HGF [239]. Following administration, NurOwn delivers these neurotrophic mediators in conjunction with the intrinsic immunomodulatory factors produced by MSCs, thereby providing a multifaceted therapeutic approach for neurodegenerative disorders.

Clinical studies have yielded encouraging findings. In a phase II trial involving patients with ALS (NCT02017912), treatment with NurOwn was associated with a reduction in disease progression compared with control subjects and demonstrated a favorable safety profile [138]. These promising results have stimulated further investigation of NurOwn and have broadened interest in its potential application to other neuroinflammatory and neurodegenerative disorders, including MS.

Despite their considerable promise, MSC priming strategies continue to face several challenges that hinder their successful clinical translation. Potential concerns include the induction of immunogenicity, increased manufacturing costs, variability in the magnitude and durability of priming effects, and the absence of standardized good manufacturing practice (GMP)-compliant protocols suitable for large-scale clinical application [240]. Furthermore, the long-term consequences of MSC priming remain insufficiently characterized, particularly with respect to safety, biological stability, and sustained therapeutic efficacy.

Addressing these limitations will require comprehensive investigations in several key areas. Future studies should systematically compare the clinical performance of different priming approaches, identify the most appropriate tissue sources for MSC isolation, and determine how priming influences epigenetic regulation, immunogenicity, and tumorigenic potential. In parallel, there is a pressing need to establish robust GMP standards and quality-control frameworks for MSC-based products, including criteria governing the production, cryopreservation, and passage-dependent characteristics of primed MSC preparations. Such efforts will be essential for ensuring the reproducibility, safety, and clinical applicability of next-generation MSC therapies.

### Genetic modification of MSCs

To further enhance their therapeutic potential, MSCs have been genetically engineered to express trophic cytokines and other beneficial gene products through the introduction of viral or non-viral expression vectors. Over the past several decades, advances in genetic engineering have enabled the generation of MSCs that secrete therapeutic peptides, growth factors, and immunoregulatory proteins, demonstrating encouraging efficacy across numerous preclinical disease models. By augmenting the intrinsic properties of MSCs, these engineered cells can exhibit enhanced regenerative, anti-inflammatory, antioxidant, and immunomodulatory functions [241].

Several examples illustrate the versatility of this approach. MSCs engineered to overexpress thioredoxin-1 (Trx1), a potent antioxidant and regulator of transcriptional and growth factor signaling, significantly improved cardiac function and reduced fibrosis in post-myocardial infarction rat models [242]. Likewise, MSCs expressing IL-12 demonstrated robust antitumor activity against multiple malignancies, including melanoma, breast cancer, and hepatocellular carcinoma [243, 244]. Similarly, IFN-γ-expressing MSCs inhibited tumor growth and reshaped the tumor microenvironment in experimental models of neuroblastoma and lung carcinoma [245, 246]. Building upon these promising preclinical findings, several genetically modified MSC-based products have advanced into clinical development, underscoring the growing potential of MSC engineering as a next-generation strategy for improving the efficacy and precision of cell-based therapies.

The advent of CRISPR/Cas9 technology has markedly advanced the genetic engineering of MSCs by enabling precise and efficient genome modification [247]. CRISPR/Cas9-mediated gene editing of MSCs has demonstrated therapeutic potential across a range of preclinical disease models. For example, gene knockdown strategies have shown efficacy in improving tissue repair and functional recovery following myocardial infarction [248]. Similarly, targeted gene knock-in approaches have been employed to enhance the differentiation capacity of MSCs, thereby increasing the availability of functionally specialized cells at sites of tissue injury [249].

The clinical feasibility of genetically modified MSCs has also begun to emerge. The TREAT-ME-1 study, a first-in-human, multicenter, open-label phase I/II trial, evaluated the safety, tolerability, and preliminary efficacy of autologous genetically engineered MSC-apceth-101 in patients with advanced gastrointestinal adenocarcinoma [250].

### Biomaterial-based strategies

Biomaterials have been extensively explored as delivery platforms for MSC-based therapies. These systems provide structural support that enhances MSC adhesion, viability, and retention at the target site while also preserving and prolonging the bioavailability of therapeutically relevant factors secreted by MSCs. By recreating aspects of the native cellular microenvironment, biomaterial scaffolds can improve cell persistence and extend the duration of therapeutic effects. Nevertheless, the implantation of biomaterials may provoke foreign-body responses (FBRs), leading to chronic inflammation, fibrotic encapsulation, and eventual failure of the implanted construct. Consequently, considerable efforts have been directed toward developing biomaterials that minimize immunogenicity and attenuate FBR-associated fibrosis [38].

Among these approaches, the incorporation of small molecule-loaded microparticles (MPs) into MSC delivery systems has shown particular promise for enhancing MSC functionality and therapeutic longevity. These MPs are composed of biocompatible materials whose properties can be precisely tailored by modifying their composition, polymer molecular weight, drug payload, and release kinetics [251]. For instance, intracellular delivery of degradable budesonide-containing MPs to MSCs resulted in an approximately fourfold increase in IDO activity in vitro compared with non-loaded MSCs, indicating a substantial enhancement of their immunomodulatory capacity [252]. This resulted in an approximately twofold increase in the ability of MSCs to suppress IFN-γ-induced activation of PBMCs, further demonstrating the potential of microparticle-based approaches to potentiate the immunoregulatory functions of MSCs [253].

MSCs are commonly delivered to target tissues using decellularized extracellular matrix (ECM)-based scaffolds, which provide a biologically relevant microenvironment that supports cell attachment, survival, and function. More recently, the development of synthetic polymers has significantly expanded the possibilities of tissue engineering and MSC delivery [254, 255]. These biomaterials can be engineered into various architectures, including porous hydrogels, sponges, membranes, and scaffold plates, allowing precise control over their physical and biochemical properties to optimize MSC retention, viability, and therapeutic performance.

### MSCs secretome as a drug delivery

Growing evidence indicates that many of the therapeutic benefits of MSCs are mediated by their secretome rather than by direct cell engraftment or differentiation. Consequently, the MSC secretome has emerged as a promising cell-free therapeutic platform capable of delivering a diverse array of bioactive molecules, including cytokines, growth factors, chemokines, extracellular vesicles, and regulatory nucleic acids. For instance, accumulating evidence indicates that MSC-EVs can recapitulate many of the beneficial effects of their parental cells and, in certain settings, may exhibit comparable or even superior therapeutic efficacy in neurodegenerative disorders [256]. Moreover, they possess several advantages over cell-based therapies in terms of safety and delivery efficiency. Their low immunogenicity and favorable immunocompatibility reduce the risk of adverse immune reactions, contributing to an improved safety profile. Furthermore, owing to their nanoscale size and intrinsic biological properties, EVs can traverse endothelial barriers, including the blood–brain barrier and blood-retinal barrier. This unique capability enables them to efficiently transport therapeutic biomolecules to the central nervous system, making MSC-EVs an attractive platform for targeted drug delivery in neurological diseases [257].

Several studies have provided encouraging evidence supporting the clinical potential of MSC-EVs. For instance, treatment with EVs derived from human placental MSCs has been associated with significant clinical improvement in patients with non-Crohn’s perianal fistulas, highlighting the therapeutic capacity of MSC-EVs to promote tissue repair and modulate local inflammatory responses [258]. In another clinical investigation, administration of WJ-MSC-EVs was associated with improved wound healing in patients with diabetes, resulting in enhanced tissue repair and overall clinical outcomes [259].

**Table 2:** Strategies to enhance the therapeutic efficacy of MSCs

Enhancement strategy	Representative approaches	Key biological effects	Advantages	Limitations
MSC priming	Hypoxia, pro-inflammatory cytokines, small molecules	↑ Immunomodulation, ↑ paracrine secretion, ↑ survival	Simple, cost-effective, improves therapeutic potency without permanent genetic alteration	Lack of standardized protocols, donor variability, transient effects, manufacturing challenges, uncertain long-term safety
Genetic engineering	CRISPR/Cas9, IL-12-MSCs, IFN-γ-MSCs, Trx1-MSCs	↑ Homing, ↑ therapeutic protein expression, ↑ differentiation, ↓ inflammation	Enables disease-specific engineering and sustained therapeutic protein expression	Potential insertional mutagenesis, off-target effects, regulatory complexity, higher production costs
Biomaterial engineering	Hydrogels, ECM scaffolds, synthetic polymers, microparticles	↑ Cell retention, ↑ engraftment, ↑ viability, controlled release	Improves cell persistence, localized delivery, and tissue regeneration	Foreign-body response, fibrosis, biomaterial biocompatibility, manufacturing optimization
MSC secretome	MSC-EVs, conditioned medium	Delivers cytokines, growth factors, miRNAs, and EVs	Low immunogenicity, no risk of uncontrolled cell proliferation, easier storage and delivery, crosses biological barriers	Lack of standardized isolation methods, batch-to-batch variability, limited large-scale manufacturing, uncertain optimal dosing

## Conclusion

The therapeutic promise of MSCs in neurological diseases is increasingly attributed to their biological modulatory properties rather than direct cell replacement. Preclinical and clinical studies across conditions such as MS, ALS, SCI, CP, PD, stroke, and Alzheimer’s disease suggest that MSCs may influence disease progression by regulating neuroinflammation, functional recovery, modulating immune responses, and promoting neuroprotective signaling pathways. BM-MSCs are the most commonly used in clinical trials, typically delivered intravenously. Other sources, such as AD-MSCs and UC-MSCs, are increasingly used because of their greater proliferative potential and lower immunogenicity. While IV injection remains the standard, alternative routes like intrathecal and intracerebral delivery are gaining attention for better CNS targeting. Research has shown that treatments involving MSCs are generally safe and patients tend to tolerate them well. However, in terms of effectiveness, results have been mixed, with studies reporting varying levels of success.

Several factors continue to constrain progress in this field, including unresolved mechanisms of action, variability in MSC sources, and challenges in optimizing delivery routes and dosing regimens. Clinical trials, though promising, are often hampered by small cohorts, open-label designs, and heterogeneity in patient populations, limiting the generalizability of findings.

While MSC-based therapies show potential for various neurological disorders, it’s crucial to consider the current stage of clinical development. Conditions like MS and SCI are the most advanced, indicated by numerous phase III trials, standardized protocols, and consistent efficacy reports. For example, intrathecal MSC delivery in progressive MS has consistently improved disability scores and neuroimaging biomarkers in phase II studies. Similarly, SCI research has shown motor and sensory function improvements with good safety across different administration methods. In contrast, uses of MSCs for PD and Alzheimer’s disease are still mainly in early, exploratory stages.

MSCs are increasingly viewed as biological modulators that primarily exert therapeutic effects via paracrine signaling, rather than through sustained engraftment. As a result, cell-free therapies, such as MSC-derived extracellular vesicles, exosomes, and secretome-based products, are gaining attention as promising options to enhance safety, scalability, and reproducibility.

To advance MSC therapies, future efforts must prioritize large-scale, randomized controlled trials with standardized protocols and rigorous biomarker validation. Innovations such as engineered MSCs with enhanced survival, hypoxia-preconditioned cells, and exosome-based delivery systems offer avenues to improve consistency and scalability. Collaborative frameworks uniting researchers, clinicians, and regulatory bodies are essential to address logistical hurdles, ethical considerations, and the translation of preclinical breakthroughs into clinically meaningful interventions. By addressing these challenges, MSC-based therapies may evolve from experimental strategies into transformative treatments, offering hope for millions affected by debilitating neurological disorders.

## Data Availability

No datasets were generated or analysed during the current study.

## Abbreviations

Aβ: Amyloid-β
ALSFRS: Amyotrophic lateral sclerosis Functional Rating Scale
AlloCB: Allogeneic umbilical cord blood
ALS: Amyotrophic lateral sclerosis
AD: Adipose tissue
APP: Amyloid precursor protein
Aβ: Amyloid plaques
BM: Bone marrow
BDNF: Brain-derived neurotrophic factor
CNS: Central nervous system
EAE: Experimental autoimmune encephalomyelitis
EDSS: Expanded Disability Status Scale
FVC: Forced vital capacity
GDNF: Glial cell-derived neurotrophic factor
GELs: Gadolinium-enhancing lesions
HGF: Hepatocyte growth factor
IDO: Indoleamine 2, 3-dioxygenase
ICH: Intracerebral hemorrhage
IGF-1: Insulin-like growth factor 1
MMPs: Matrix metalloproteinases
MSCs: Mesenchymal stem cells
miRNAs: microRNAs
MS: Multiple sclerosis
NET: Neutrophil extracellular trap
NSCs: Neural stem cells
NO: Nitric oxide
NGF: Nerve growth factor
NF-L: Neurofilament light chain
NFTs: Neurofibrillary tangles
PGE2: Prostaglandin E2
PD: Parkinson’s disease
ROS: Reactive oxygen species
SCI: Spinal cord injury
TBI: Traumatic brain injury
TNT: Tunneling nanotube
T25FW: Timed 25-foot walk
UC: Umbilical cord
VEGF: Vascular endothelial growth factor
